# Characteristic functional cores revealed by hyperbolic disc embedding and *k*-core percolation on resting-state fMRI

**DOI:** 10.1038/s41598-022-08975-7

**Published:** 2022-03-22

**Authors:** Wonseok Whi, Youngmin Huh, Seunggyun Ha, Hyekyoung Lee, Hyejin Kang, Dong Soo Lee

**Affiliations:** 1 Department of Molecular Medicine and Biopharmaceutical Sciences, Seoul National University, Seoul, South Korea; 2 Department of Nuclear Medicine, Seoul National University and Seoul National University Hospital, Seoul, South Korea; 3 Medical Research Center, Seoul National University, Seoul, South Korea; 4 Division of Nuclear Medicine, Department of Radiology, Seoul St. Mary’s Hospital, College of Medicine, The Catholic University of Korea, Seoul, South Korea; 5 Biomedical Research Institute, Seoul National University Hospital, Seoul, South Korea

**Keywords:** Cognitive neuroscience, Computational neuroscience, Complex networks, Phase transitions and critical phenomena, Applied mathematics

## Abstract

Hyperbolic disc embedding and *k*-core percolation reveal the hierarchical structure of functional connectivity on resting-state fMRI (rsfMRI). Using 180 normal adults’ rsfMRI data from the human connectome project database, we visualized inter-voxel relations by embedding voxels on the hyperbolic space using the ${\mathbb{S}}^{1} /{\mathbb{H}}^{2}$ model. We also conducted *k*-core percolation on 30 participants to investigate core voxels for each individual. It recursively peels the layer off, and this procedure leaves voxels embedded in the center of the hyperbolic disc. We used independent components to classify core voxels, and it revealed stereotypes of individuals such as visual network dominant, default mode network dominant, and distributed patterns. Characteristic core structures of resting-state brain connectivity of normal subjects disclosed the distributed or asymmetric contribution of voxels to the *k*_max_-core, which suggests the hierarchical dominance of certain IC subnetworks characteristic of subgroups of individuals at rest.

## Introduction

The brain is a high-dimensional complex and integrated network that is composed of multiple modular and specialized networks, distributed spatially, and combined to form a multimodular structure^1–5^. The conundrum of how these modules are aggregated to form a single coherent network with preserved functionality remains a fundamental question for unveiling the functional architecture of the human brain in the resting state and upon activation^6,7^. Individual differences add complexity to a succinct understanding of this question.

Recent works in network science suggest that the aggregation of these modules is facilitated by a set of essential voxels that integrate intramodular and intermodular information throughout the network^2,3,8,9^. Essential nodes were initially supposed to be hub nodes with high degrees or high centrality on brain graphs but were soon suggested to be core influencer nodes with a wide range of initial degrees on decomposition^8^. In physical networks, disruption in these core nodes leads to abrupt disintegration, which is called network dismantling or targeted damage^10–13^. Disruption in the brain graph is associated with serious neuropsychiatric diseases with disrupted associative functionality^14,15^.

Therefore, it is important to identify which nodes compose the core structure, and if ever the resting state core is individually unique, then their individual differences in the core composition should be disclosed using voxel-based representation of brain graphs. Recent studies of mathematics and neurosciences have accomplished this job successfully with physical networks and probably with the brain^3,8,12–16^, implementing hubness, centrality measures such as degree, betweenness, eigenvector, and leverage centrality^17–20^, or *k*-core^21–25^. The *k*-core percolation describes the architecture of the backbones of the network by filtering out peripheral nodes and searching for remaining central nodes, where the coreness, *k*, acts as the threshold for sustaining node connectivity along the filtration. The *k*-core percolation was used to understand the forward (phase transition) and backward (k-decomposition) behavior of networks, and brain networks can be dissected in a similar way as was done for graph filtration thresholding^26–30^.

In our previous work, we addressed the problem of difficulty in visualization and thus formed mental imagery of the object brain graphs by considering their geometric characteristics. We adopted an analytical framework for visualizing a complex, multimodular network for functional brain networks with scale-freeness by embedding the networks into the latent geometric model of ${\mathbb{S}}^{1} /{\mathbb{H}}^{2}$.

Based on the Popularity $\times$ Similarity optimization model^31^, the geometric meaning of ${\mathbb{S}}^{1} /{\mathbb{H}}^{2}$ model is that the angular coordinates (described by ${\mathbb{S}}^{1}$ counterpart) correspond to the similarity along nodes, in the manner that the closely connected nodes are clustered in similar angular coordinates, while degrees correspond to the popularity of node in the network, so that the radial coordinates (described by ${\mathbb{H}}^{2}$ counterpart) account for the popularity (degree) of node, as the popular nodes are located closer to the origin.

The model has been successful in revealing the hidden geometry of many other real complex networks of non-Euclidean nature^31–33^. The embedding of the network into the geometric model was performed by using the software named *Mercator*, introduced by García-Pérez et al.^33^, which makes use of Laplace eigenmaps (LE) for the reduction of dimension and maximum likelihood estimation (MLE) techniques for acquiring the most appropriate geometric object on hyperbolic discs representing the original network with fidelity^31–34^. This solved two issues: (1) 2-dimensional representation of complex brain graphs with flexible annotation of functionally cooperating voxel groups and (2) thresholding to sort out necessary edges to make the complex brain graphs obey power law and thus scale-freeness^34^, which implies the self-similarity and heterogeneity of degree distribution. This shares some topological features with functional brain network, and compatible with hyperbolic embedding of the network^31–34^.

In this work, we analyzed functional brain networks from healthy human young adults by analyzing rsfMRI data and visualized functional subnetworks, i.e., independent components (ICs), using hyperbolic embedding. Then, we investigated how each functional subnetwork was composed of the subset of voxels with high core-ness, revealed by *k*-core percolation as a measure of centrality. We characterized the *k*_max_-core voxels for their degree distribution belonging to each IC, showing the plausible influencer behavior of these IC subnetwork voxels upon *k*-core percolation eventually to find which subnetworks are the dominant by counting the voxels belonging to them at rest in normal individuals. We asked whether the individuals had common or characteristic core structures in terms of their *k*_max_-core IC-voxel compositions.

## Results

### Method of hyperbolic embedding of voxels on individual rsfMRI

**Figure 1:**
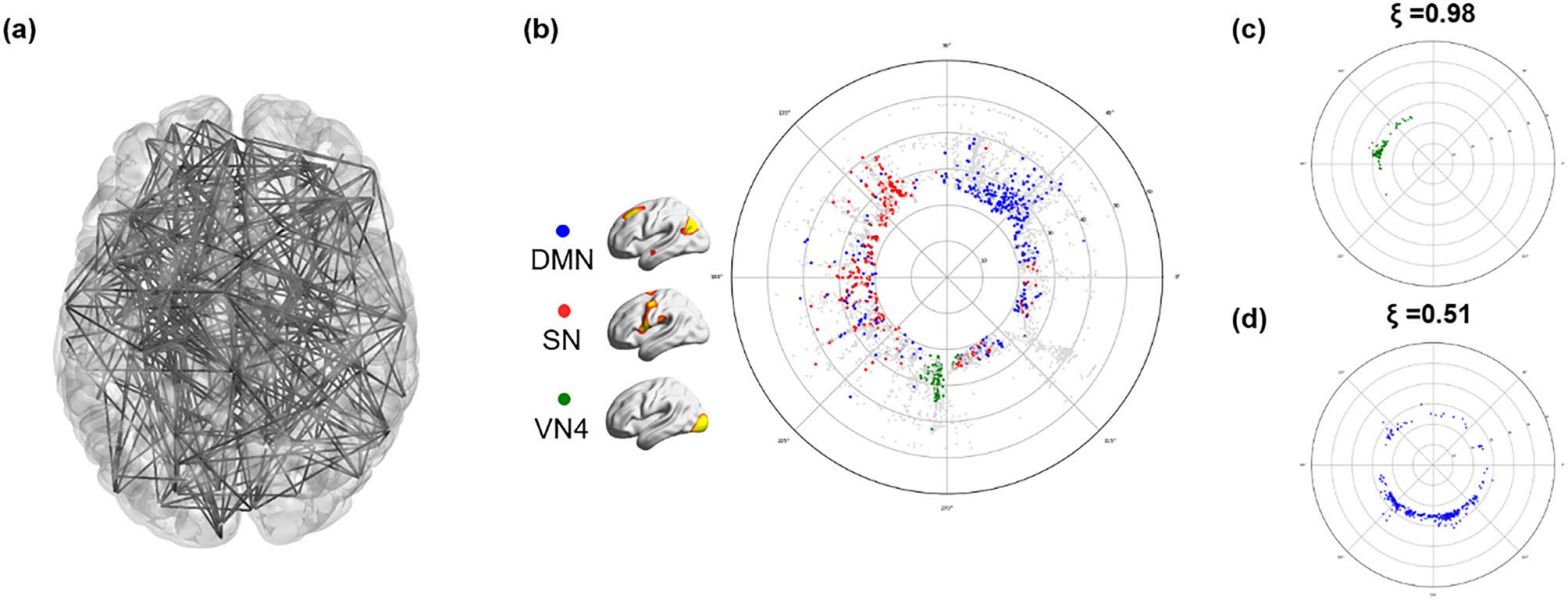
Hyperbolic disc embedding and angular coherence of the voxels on the disc. (**a**) A brain network with 500 random nodes was displayed using 3-dimensional MNI coordinates projected on a 2-dimensional brain space. This visualization provides intricate edges and nodes that are not easily discernible. (**b**) Hyperbolic disc embedding provides easy-to-recognize visualization of the voxels on the hyperbolic disc. In this hyperbolic disc, 5937 voxels were used, which shows the intervoxel relationships between voxels in an unoverlapped way with 10 times more voxels than the one in (**a**). Specifically, the brain was resampled into 5937 6 × 6 × 6 mm^3^ voxels, which were assigned as voxels of subnetworks belonging to independent components (ICs)^34^. The hyperbolic distance between two voxels on this hyperbolic disc is equivalent to the correlation proximity between these voxels in Euclidean space. The radial coordinate responds to the degree of the voxel, i.e., the hub voxel is near the center of the disc, and the angular coordinate responds to the similarity of voxels^32^. As an example, voxels from the independent component (IC) subnetworks are presented in different colors. The voxels from the salience network (SN) (red) are more widely distributed on angular coordinates than the default mode network (DMN) (blue) or visual network 4 (VN4) (green). (**c**) The angular coherence quantifies the degree of aggregation of a group of voxels based on coordinates. It ranges from 0 to 1, and a higher value indicates compact gathering with smaller differences in angles between voxels in the group. Widely spread voxels have a lower value of angular coherence. The angular coherences of voxels comprising (**c**) VN4 (ξ = 0.98), (**c,d**) DMN (ξ = 0.51) are shown.

To visualize the correlation structure of the voxel composition of the complex functional brain network, we adopted a method to transfer the high-dimensional connection (edge) information to the hyperbolic disc space. According to our previous investigation^34^ that looked for an optimal non-Euclidean space for embedding the intervoxel correlation structure, we simply chose 2-dimensional hyperbolic disc embedding. Hyperbolic disc representation reflected the original high-dimensional edge information similarly well to the high-dimensional Euclidean embedding alternatives^34^. To the best of our knowledge, the high-dimensional correlation structure rendered by hyperbolic disc embedding serves an easy-to-recognize visibility, which is very difficult to achieve via Euclidean 2-dimensional representation (Fig. 1a,b).

Unlike our previous study, which used anatomically predefined regions^34^, we used voxel correlation to visualize intervoxel relationships for hyperbolic disc embedding using the ${\mathbb{S}}^{1} /{\mathbb{H}}^{2}$ model^33^. The output easily disclosed the belonging characteristics of the voxels to ICs on a hyperbolic polar coordinate. Edge weight on the voxel-voxel correlation matrix was thresholded to yield the adjacency matrix after confirming the scale freeness of the resultant degree distribution of the voxels while preserving the size of the largest component as large. This allowed us to confirm the power law of its degree distribution and scale freeness to be fit for the hyperbolic model. Reproducibility on repeated embedding was tested in an exemplary case with repeated embedding with the Mercator algorithm^33,34^ (Supplementary Fig. 1).

### Hyperbolic disc embedding of rsfMRI voxels and their belonging to IC subnetworks

Using 180 Human Connectome Project subjects’ data, we produced intervoxel correlation networks with 5937 voxels. Raw voxels were downsampled to yield computationally plausible size, but we still called the enlarged units of aggregates of voxels. The correlation coefficients between two voxels were calculated to define the edges of the network. These networks were binarized after confirming the linearity on a log–log plot of the degree distribution of the output adjacency matrix, and the largest components of the network having at least 80% of the entire 5937 voxels were embedded on the hyperbolic discs using the previously described method^34^. Embedding was performed on the hyperbolic disc using the ${\mathbb{S}}^{1} /{\mathbb{H}}^{2}$ model according to previously reported methods^33,34^.

The adjacency matrix was then converted to fit into the ${\mathbb{S}}^{1} /{\mathbb{H}}^{2}$ model finally to yield the polar coordinates for the voxel in hyperbolic disc. Using the polar coordinates, we estimated angular coherence to investigate how the embedded voxels were angularly similar. After successful embedding using Mercator^33^, voxels included in the largest components were more than 80% (5391 ± 224 voxels), with edges ranging from 274,634 to 3,894,033 (1.6–22% of possible edges). A randomly sampled case was repeatedly embedded in this ${\mathbb{S}}^{1} /{\mathbb{H}}^{2}$ model, and their reproducibility is shown in Supplementary Fig. 1 and Supplementary Table 1. Voxel-based embedding in this study yielded a similar feature of reproducibility. We confirmed that 2-dimensional hyperbolic disc embedding using the ${\mathbb{S}}^{1} /{\mathbb{H}}^{2}$ model was feasible using voxel-based data and analyzed the pattern of embedded voxel-voxel relationships. We used only the positive correlation and the negative correlation left for the following study, including the interdependent multilayered characteristics of brain networks on hyperbolic disc embedding.

This embedding provided clearer visibility of intervoxel relations on 2-dimensional space (disc) than any conventional method of visualization (Fig. 1a,b). Inspired by the initial suggestion of using hyperbolic discs for popularity and similarity representation of growing complex networks and translation of this method to the ${\mathbb{S}}^{1} /{\mathbb{H}}^{2}$ model, the derived network was described in a hyperbolic disc, where the angular coordinates are used as proxy variables revealing similarity of nodes, while the nodes with higher popularity (degree) are placed closer to the center of disk^33^. This enabled us to assume that the angular coherence on the hyperbolic disc reveals similarity of the group of voxels and that the closer distance of a voxel to the disc center represents a higher degree with greater popularity^34^. We identified the voxels belonging to specific ICs (fifteen ICs) obtained from conventional group ICA performed in all 180 subjects^35,36^.

### Angular coherence of hyperbolic disc-embedded voxels belonging to IC subnetworks

We investigated the distribution pattern of voxels on the hyperbolic discs and their belongings to each IC among normal individual subjects. Group ICA annotated each voxel to its IC. Angular coherence of grouped voxels according to ICs was measured on embedded hyperbolic discs. When voxels belonging to an IC were grouped closely together within narrow angles from the disc center, the IC and its voxels were called to have higher proximity with higher angular coherence (ranging from 0 to 1) (Fig. 1c,d). Angular coherence of ICs represented how close the voxels in an IC gathered together as a subnetwork. Subnetworks were labeled in two ways: one with the functional label of the voxels to the 15 ICs reminded the multiscale renormalization of brain graphs^37^ (Supplementary Fig. 2). Another anatomical label used 15 predefined lobes based on the Brainnetome atlas^38^ (Supplementary Fig. 3).

**Figure 2:**
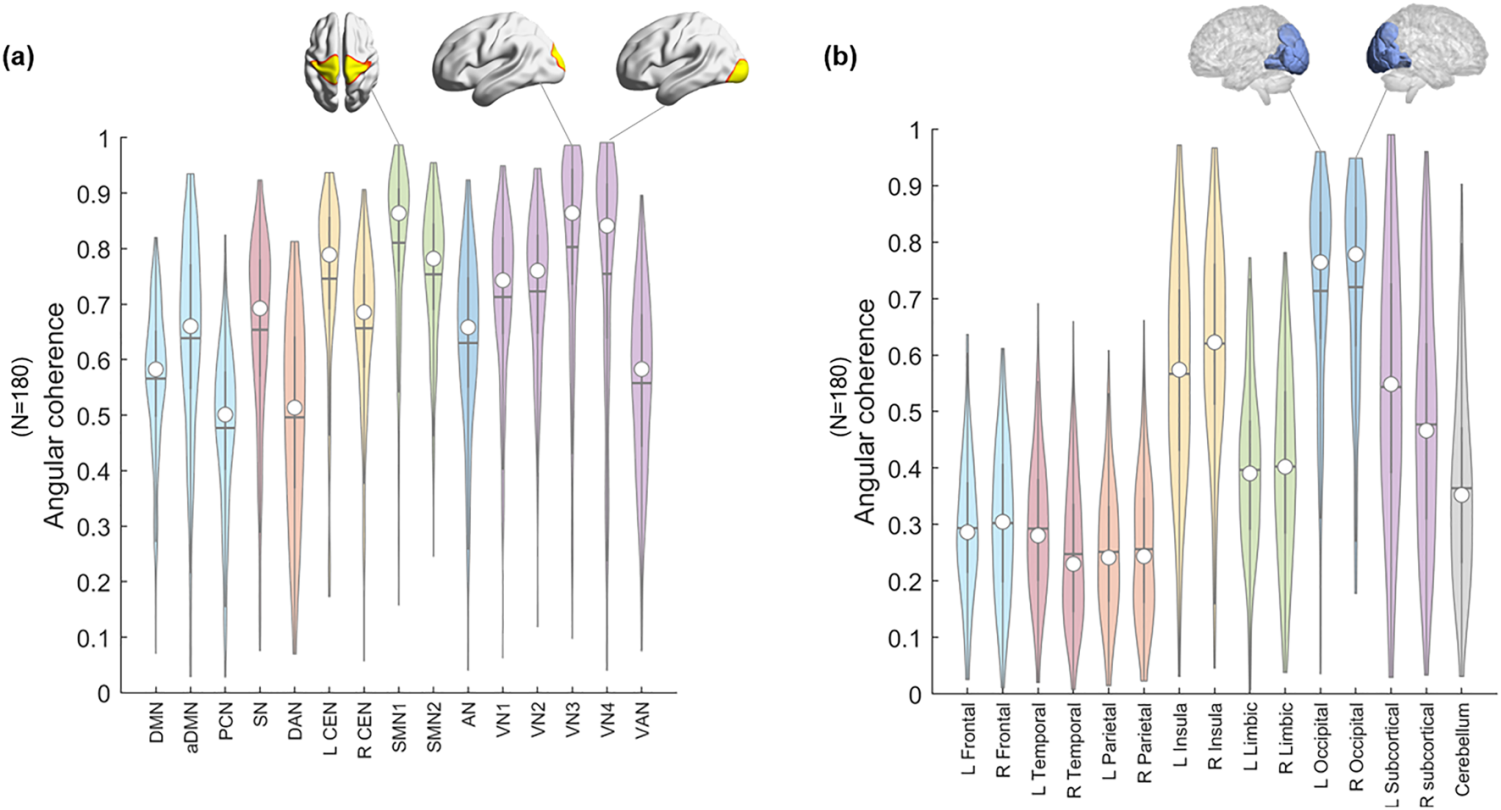
Distribution of the angular coherences of 180 individuals’ voxels on hyperbolically embedded discs. Groups of voxels belonged to (**a**) functional labels derived from group independent component analysis (ICA) and (**b**) atlas-based anatomical labels. (**a**) Fifteen independent components were chosen from group ICA for the entire data: default mode network (DMN), anterior DMN (aDMN), precuneus network (PCN), salience network (SN), dorsal attention network (DAN), left central executive network (L CEN), right CEN (R CEN), sensorimotor network (SMN) 1/2, auditory network (AN), visual network (VN) 1/2/3/4, and visual attention network (VAN). The spatial maps of ICs were binarized (Z > 6), and voxels were classified to belong to each of the specific ICs. The coordinates of groups of voxels per specific IC were calculated (see “Methods” section). The values of angular coherence of SMN and VN3 and VN4 were the highest. (**b**) The whole brain was segmented into fifteen anatomical lobes based on the Brainnetome atlas to yield anatomical labels: bilateral frontal/temporal/parietal/limbic/occipital/subcortical and a cerebellum. The coordinates of groups of voxels per lobe were calculated (see “Methods” section). The median of each distribution is indicated with a circle, and the mean is indicated with a horizontal line.

For the 180 subjects (Supplementary Table 2), the angular coherence calculated using functional labels tended to be higher than that calculated using anatomical labels. In the functional label, sensorimotor network 1 (SMN1) (~ 0.81) and VN3 (~ 0.80)/5 (~ 0.75) showed the highest angular coherence, and the precuneus network (PCN) (~ 0.48) and dorsal attention network (DAN) (~ 0.50) showed the lowest angular coherence (Fig. 2a, Supplementary Table 3). Bilateral occipital lobes (left: ~ 0.71, right: ~ 0.72) showed the highest angular coherence, and parietal (left: ~ 0.25, right: ~ 0.26) and temporal lobes (left: ~ 0.29, right: ~ 0.25) showed the lowest angular coherence in the anatomical label (Fig. 2b, Supplementary Table 4).

The number of voxels belonging to the 15 ICs ranged from 158 (anterior default mode network (aDMN)) to 443 (visual network (VN) 2) (Supplementary Table 3). On the hyperbolic disc, the angular coherence ($\upxi )$ of the DMN was 0.57 ± 0.14 (n = 180) and that of VN1 was 0.71 ± 0.15. In an individual chosen, for example, VN4 (green circle) voxels had $\upxi =$ 0.95, salience network (SN, red circle) $\upxi =$ 0.52 or the default mode network (DMN, blue circle) $\upxi$ = 0.50. The voxels embedded on the hyperbolic disc disclosed their own unique pattern but also revealed the common distribution characteristics. The hyperbolic disc should be read with polar coordinates with its intervoxel distance on a logarithmic scale in the radial direction and hyperbolic contribution of the intervoxel angle in arc hypercosine to the distance (see “Methods” section). Evidently, the rotation/reflection symmetry^39^ of this embedded disc representation and other symmetries, such as branch permutation related to the hyperbolicity of the disc, should be considered in the interpretation of voxel distribution^40^.

Regarding laterality, the angular coherence of each left and right hemisphere based on the Brainnetome atlas, except cerebellum, showed no significant difference (Supplementary Fig. 4a). An analysis using anatomical labels, temporal lobes, and subcortical regions showed significantly different angular coherence between the left and right sides (*p* < 0.05, Supplementary Fig. 4b).

### Discovery of k_max_-core voxels on k-core percolation and its visualization on hyperbolic embedded discs

Using the adjacency matrix obtained by scale-freeness guaranteed thresholding, we proceeded to find the core voxels. We asked which voxels in the IC subnetworks survived the decomposition by *k*-core percolation. We questioned whether the hub voxels with higher degrees would remain solely or whether other voxels with fewer edges would join the survivors. Which voxels belong to which ICs would remain and dominate or participate as core voxels at the end of *k*-core percolation.

**Figure 3:**
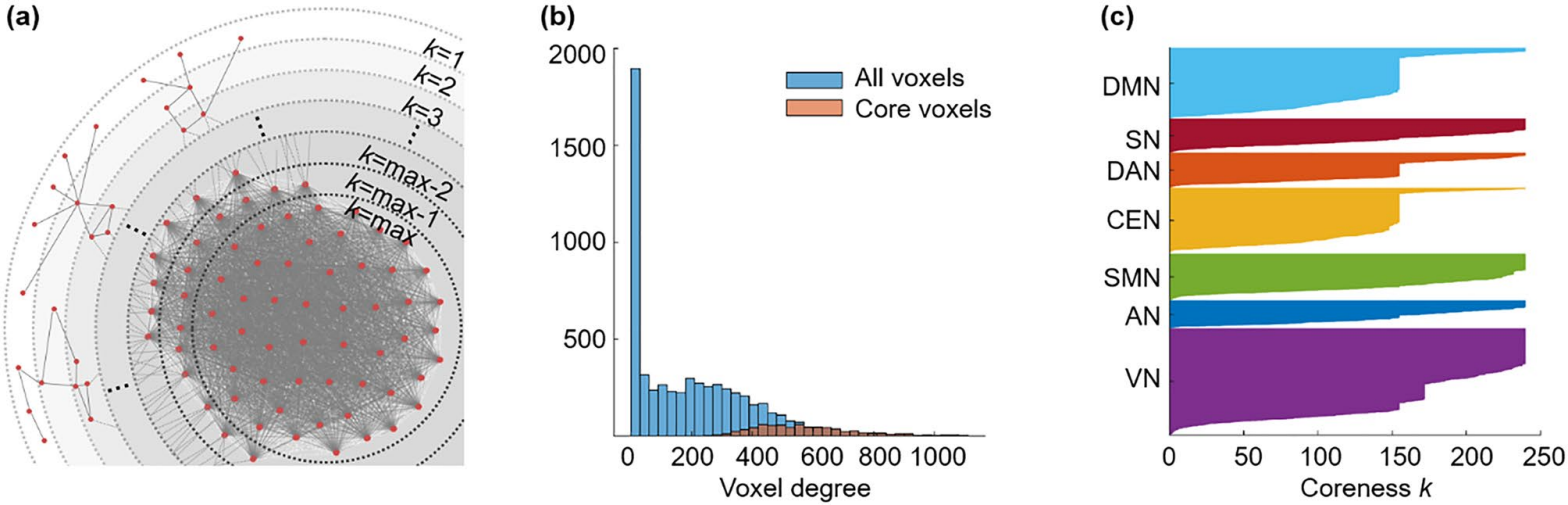
Conceptual illustration of *k*-core percolation and plots describing *k*-cores and the *k*_max_-core derived by *k*-core percolation. (**a**) *k*-core percolation renormalizes the brain network by peeling the layers with *k*-steps from *k* = 1 to *k* = *k*_max_ for the brain network. Intervoxel correlations were thresholded to yield an adjacency matrix after checking the scale freeness of the degree distribution of voxels and put into hyperbolic disc embedding and *k*-core percolation. The voxels with a degree equal to coreness *k* are eliminated, and recalculation of the voxels’ degree proceeds to the next step and continues until the remaining voxels forming the largest component at that step are disintegrated into many pieces at once. The voxels at this step *k* = max are called *k*_max_-core voxels. (**b**) The *k*_max_-core voxels included not only the voxels with the largest degree on the initial adjacency matrix but also the voxels with smaller degrees. This histogram shows the degree distribution of voxels from one subject (#100,206). The blue bins represent all the voxels, and the red bins represent *k*_max_-core voxels. *k*_max_ was 240, and the degrees of *k*_max_-core voxels ranged from 260 to 1088. The *k*-core percolation finds *k*_max_-core voxels that have dense connectivity among themselves as well as hierarchically at the apex within their belonging independent components (ICs) and even the voxels with lower down to one-fourth of voxel with the highest degree. (**c**) A flag plot shows the changing *k*-cores of a subject that vary with the coreness *k* value during *k*-core percolation. Each voxel that belongs to a specific IC is shown on the y-axis, and the voxels comprising each *k*-core are colored. This subject has a *k*_max_ core with a 240 k-value and shows the first abrupt decrease during *k*-core percolation in DMN, DAN, CEN, and VN (*k* ≈ 156) and the second abrupt decrease in VN (*k* ≈ 172).

**Figure 4:**
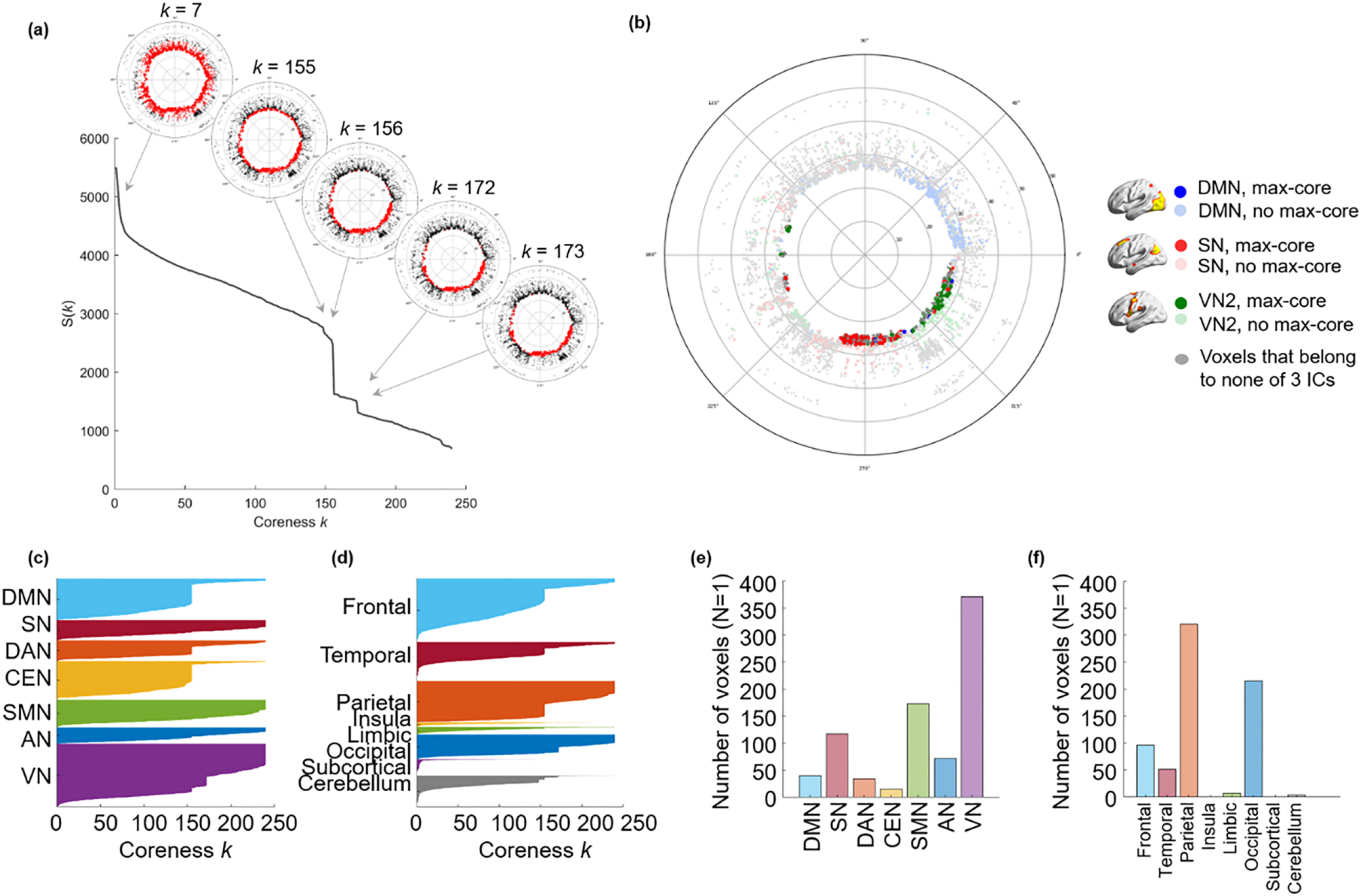
The *k*-cores and the *k*_max_-core depicted by flag plots and hyperbolically embedded discs. Each individual has his/her own size of *k*_max_-core and changes in the size of *k*-cores according to coreness *k* during *k*-core percolation. In individuals, a few abrupt decreases were observed over the gradual change of the largest component. (**a**) The coreness *k* and the size of the core S (*k*) of a subject were plotted, showing two abrupt changes. Specific *k*-cores that showed an abrupt decline (*k* = 7, 155, 156, 172, 173) were embedded on the hyperbolic discs to show the explosive decrease in core voxels. The voxels belonging to a *k*-core are denoted with red circles; otherwise, they are denoted with black circles on these hyperbolic discs. When the plot shows an abrupt decrease in S (*k*), voxels belonging to the *k*-core are reduced at once. (**b**) In an individual, *k*_max_-core shows the various sizes and independent component (IC)-voxel compositions. The *k*_max_-core (*k* = 240) of a subject is presented as an example. There were 694 voxels left on the *k*_max_-core, and the voxels that belonged to the default mode network (DMN) were in blue, salience network (SN) in red, and visual network 3 (VN3) in green. Voxels other than *k*_max_-core voxels are in pale circles. (**c**) The components of each *k*-core from one subject that vary with coreness* k* value are shown on the flag plot using functional IC labels (**c**) and anatomical labels (**d**) that annotate voxels to specific subnetworks. In the flag plot, every voxel is presented on the y-axis with labeling, and the horizontal bar of each voxel refers to the maximum *k* of *k*-cores to which the voxels belong. The voxels from each subnetwork on the y-axis were sorted in descending order of *k*. The bar plots show the affiliation of *k*_max_-core voxels (**e,f**). This individual showed abrupt declines in *k*-core size in the DMN, dorsal attention network (DAN), central executive network (CEN), and VN by functional labels and in the frontal, temporal, parietal, and occipital lobes by anatomical labels. The *k*_max_-core voxel was classified using larger functional labels (7 ICs) (**e**) and anatomical labels (8 lobes) (**f**). In this individual, VN or parietal/occipital lobe voxels belonged in the largest number to the *k*_max_-core.

During *k*-core percolation, voxels with a degree *k* were designated as *k*-shell and removed, and *k* started from 1 with an increment of 1. This *k-*shell removal was accompanied by recalculation of the remaining voxels’ degrees and was repeated until the step of *k*_*max*_; this is the maximum *k* such that *k*-core is not empty. After an increment of the value of *k* by one, no voxel remains in the *k*-core. This procedure peels the layers of a network based on the n-degree of voxels. The voxels with a degree equal to the coreness *k* are called *k*-core, and voxels with the highest degree at the step of coreness *k*_max_ are called *k*_max_-core (Fig. 3a,c). The *k*_max_-core voxels were not always the voxels with the highest degree at the beginning (Fig. 3b) since *k*-core percolation sequentially eliminated voxels with lower degrees than *k* and recalculation made voxels survive or not with their remaining connections with then-survivors (Fig. 4).

When *k*-cores derived from *k*-core percolation were embedded on a hyperbolic disc, as *k* increased, voxels farther from the center of the disc were removed earlier, and then voxels near the disc center tended to remain in the *k*-cores. Finally, survivors composed *k*_max_-core (Fig. 4a,b, Supplementary Figs. 5, 6). The core voxels had higher degrees and strong inter-connections. Hence, they tended to locate near the center of the hyperbolic discs, and it represented higher popularity surviving k-core percolation and hierarchically higher level of the networks.

**Figure 5:**
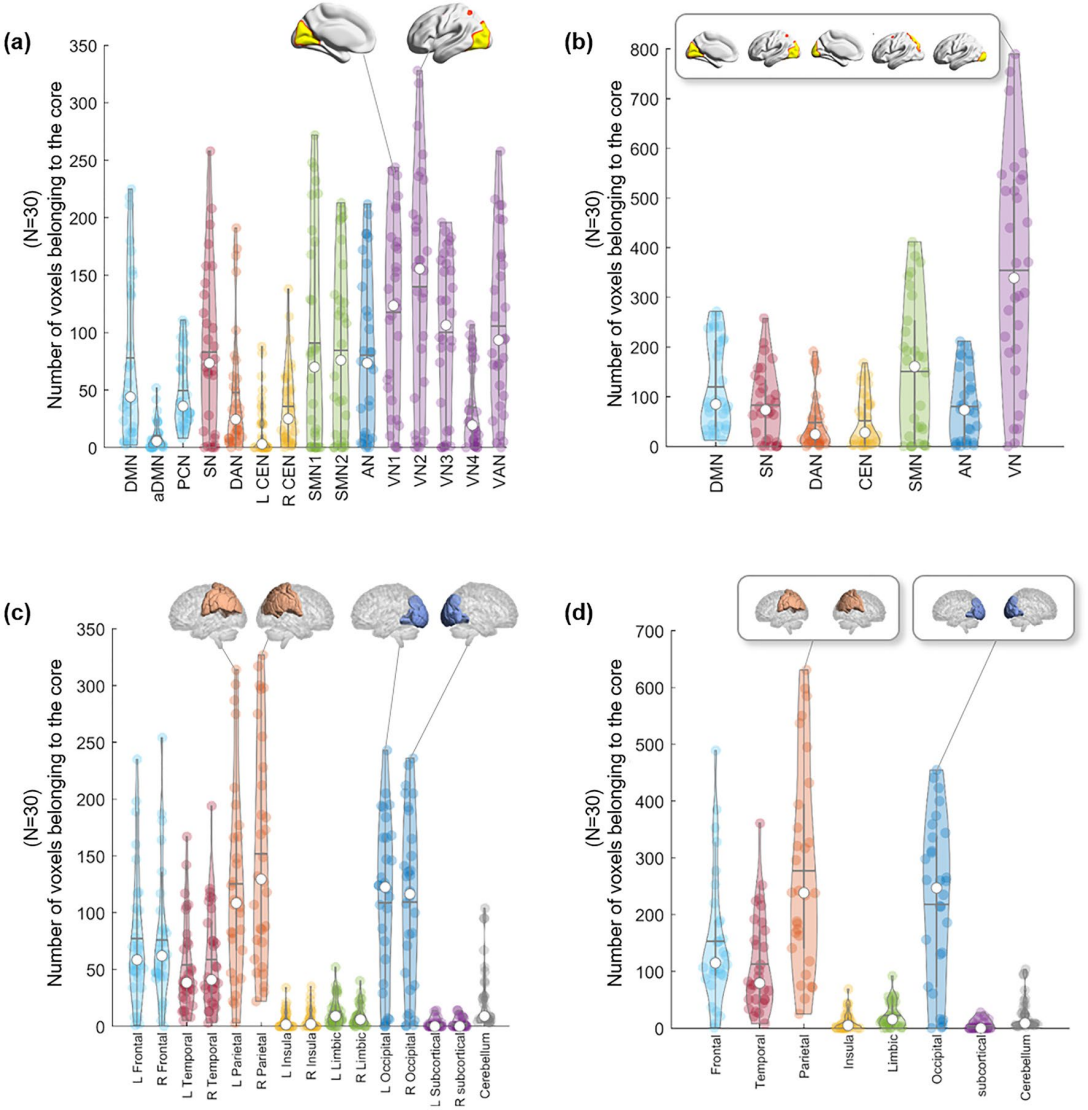
The plots show which subnetworks, in 30 individuals, the *k*_max_-core voxels belong to. *k*-core percolation yielded *k*_max_-core voxels for each individual and which independent components (ICs) or lobes those *k*_max_-core voxels belonged. ICs were represented as 15 (**a**) or as the seven categorized (**b**). Five visual networks (VN1/2/3/4, VAN) into one visual network (VN), etc. Since some of the voxels belong to multiple ICs, categorized VN had slightly fewer voxels than the sum of the number of voxels of constituents (V1/2/3/4, VAN). (**a**) According to the fifteen functional labels, visual subnetworks, VN1, VN2, and VN3, and the visual attention network (VAN) were leading in the number of voxels among *k*_max_-core voxels, followed by sensorimotor networks (SMN 1, 2) and the salience network (SN). (**b**) Once categorized, the propensity of VN among the seven was outstanding. (**c**) Anatomical labels for both lobes and cerebellum showed prominence of occipital and parietal lobes among the fifteen, and (**d**) once categorized to eight, parietal and occipital lobes were sustained.

Due to the varying number of edges at the beginning, the number and location of *k*_max_-core voxels varied greatly from individual to individual (mean number of voxels at *k*_max_-core: 826 ± 503, range 251–1854). We counted how many *k*_max_-core voxels belonged to specific resting-state IC/lobe subnetworks (Fig. 5). According to the ICs, VN1 (range 0–244)/VN2 (range 0–328)/VN3 (range 0–196) and the visual attention network (VAN) (range 0–258) showed the largest mean number of voxels comprising *k*_max_-core (Fig. 5a, Supplementary Table 5). Once combining ICs into seven networks, VN (sum of VN1, 2, 3, 4 and VAN) (range 0–790) had the largest number of *k*_max_-core voxels, and DAN (range 0–191) had the smallest (Fig. 5b, Supplementary Table 6). According to the lobes, the left (range 22–327) and right (range 3–314) parietal and left (range 0–236) and right (range 0–243) occipital lobes had the largest number of *k*_max_-core voxels (Fig. 5c, Supplementary Table 7). Using eight-category anatomical labels, parietal (range 25–631) and occipital (range 0–455) voxels had the largest *k*_max_-core voxels (Fig. 5d, Supplementary Table 8).

### Voxel-subnetwork composition of k_max_-core and their subnetwork distribution pattern among individuals

Voxels remaining at the last step of k-core percolation were annotated to the ICs or the lobes, and their initial degrees were rendered as histograms, which revealed the degree distribution of the *k*_max_-core voxels ranging from 187 to 3847 or from 21 to 52% (the percent of the degree of each *k*_max_-core voxel per the degree of the voxel with the highest degree). Interestingly, the distribution of the degrees of the *k*_max_-core voxels varied between individuals who showed a spectrum in the distribution of the dominance (or nondominance, meaning even participation of voxels in *k*_max_-core) of the ICs to which the *k*_max_-core voxels belonged (Supplementary Figs. 7–11).

**Figure 6:**
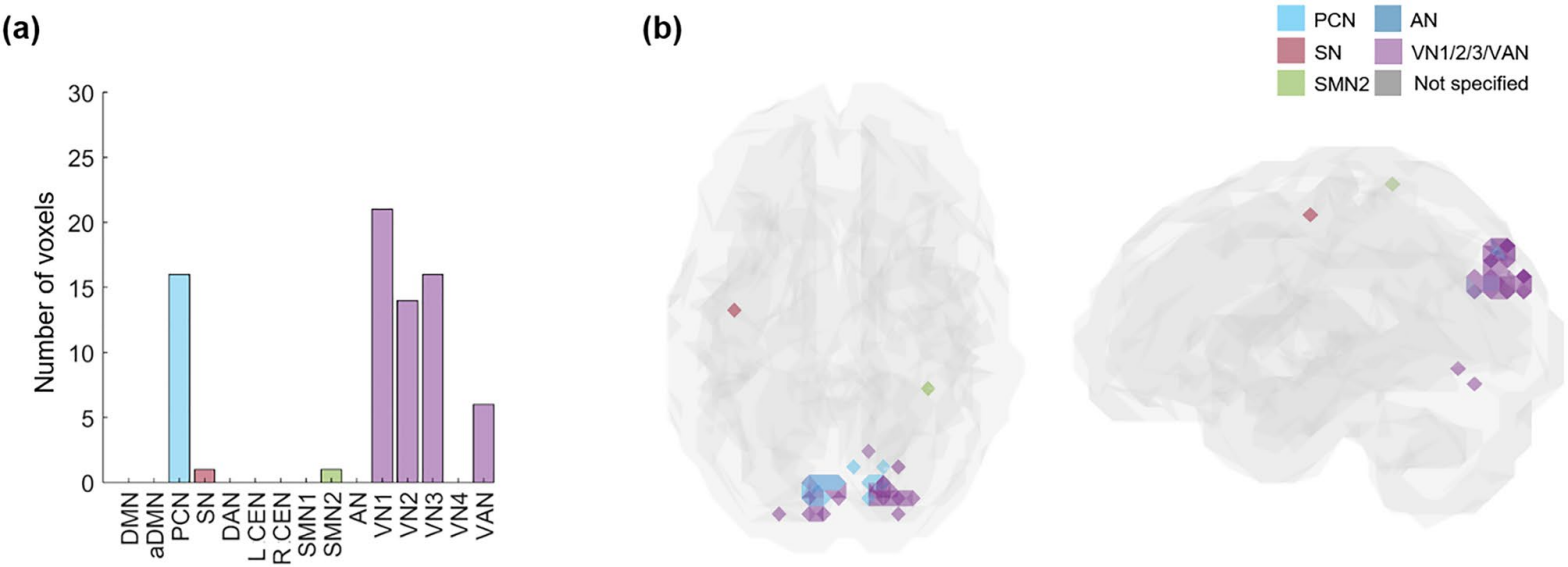
The common core voxels were shared by 60% of individuals. *k*-core percolation disclosed to which IC subnetworks prevalently among individuals, the *k*_max_-core voxels belonged. (**a**) A bar plot showing the affiliation of *k*_max_-core voxels. Shared voxels of the precuneus network (PCN), visual network 1 (VN1), VN2, V3, and visual attention network (VAN) were easily found and rare voxels in salience (SN) and sensorimotor network 2 (SMN2). (**b**) The shared voxels on the template brain were visualized. Voxels of *k*_max_-core are found in VN and PCN.

More specifically, VN included the largest number of *k*_max_-core voxels in 73% of subjects (22 among 30) than any other resting-state IC subnetwork, and more than half of the *k*_max_-core voxels belonged to VN in 40% of subjects (12 among 30). The degrees of *k*_max_-core voxels belonging to VN ranged from high to low values, similar to the degrees of the voxels belonging to the other ICs, such as DAN, DMN, and SN (Supplementary Figs. 10, 11). Over the individual differences, we questioned whether there was any group-level *k*_max_-core and found that 34 *k*_max_-core voxels were shared in common by more than 60% of subjects (18 among 30), and VN occupied the largest number of these common *k*_max_-core voxels (Fig. 6a,b). VN could be said to be the most dominant IC subnetwork (V1: 21, V2: 14, V3: 16, and VAN: 6 voxels). In addition to VNs, PCN included the largest number of *k*_max_-core voxels (commonly shared in 60% of subjects: 16 voxels) (Fig. 6a,b). When the anatomical label was applied, 34 *k*_max_-core common voxels in 60% of subjects (n = 18) mainly belonged to occipital and parietal regions. In more detail, 17 voxels in bilateral lateral superior occipital regions, 13 voxels in the bilateral ventromedial occipital regions, three voxels in the parietal regions (superior parietal lobule, precuneus, postcentral gyrus), and one in the frontal region participated in the *k*_max_-core. We suggest that posterior area voxels, including VN/PCN or occipitoparietal regions, are candidates for the common core in more than half of normal subjects to preside over their hierarchically lower voxels in the same ICs and the voxels belonging to the other ICs or lobes.

**Figure 7:**
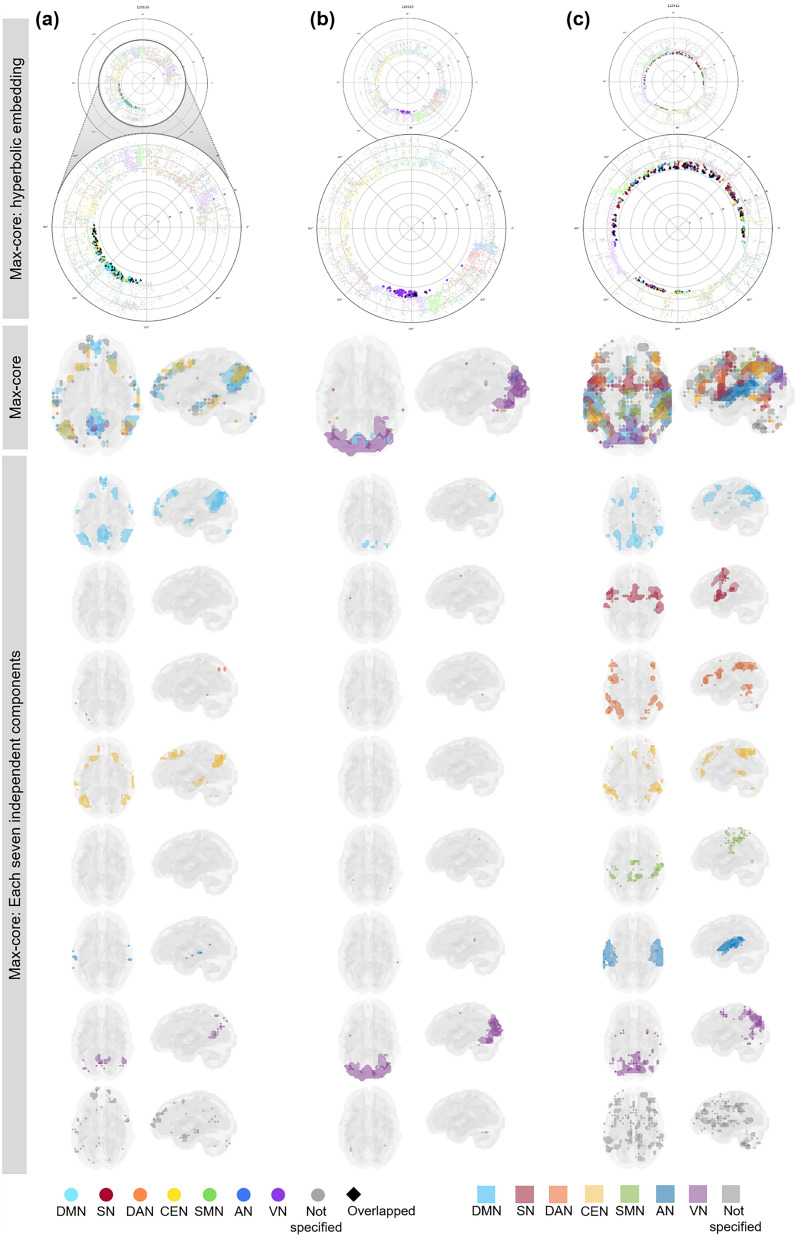
Three types of *k*_max_-core voxel-independent component (IC) compositions at the end of *k*-core percolation. There were three types, named based on which ICs the *k*_max_-core voxels belong: VN-dominant, DMN-dominant and distributed. Categorized functional labels, consisting of the default mode network (DMN), salience network (SN), dorsal attention network (DAN), central executive network (CEN), auditory network (AN), and visual network (VN), were used to classify *k*_max_-core voxels according to their belonging to these categorized labels. In the top row, each individual’s* k* max core was embedded on the hyperbolic disc. The *k*_max_-core was enlarged and shown in detail. In the middle, *k*_max_-core was visualized on the 3-dimensional brain. At the bottom, the *k*_max_-core voxels belonging to seven networks are shown separately. (**a**) Individuals with more than 40% of their *k*_max_-core voxels in the DMN were classified as DMN-dominant. *k*_max_-core voxels of one example (129,533) of the DMN-dominant type show blue regions indicating *k*_max_-core voxels that belong to the DMN. More than 60% of *k*_max_-core voxels were in DMN regions ranging over the precuneus, lateral parietal cortex, and medial prefrontal regions. (**b**) Individuals with more than 40% of *k*_max_-core voxels being in VN were classified as VN-dominant. A VN-dominant individual (126,325) shows that more than 80% of *k*_max_-core voxels belong to the VN, ranging over medial and lateral occipital and parietal regions. (**c**) Individuals were classified as having a distributed pattern when no dominant IC subnetworks were found. In an example case (110,411), every subnetwork voxel contributed to less than 20% of *k*_max_-core voxels. We counted all duplicates when the *k*_max_-core voxels belonged to multiple IC subnetworks.

**Figure 8:**
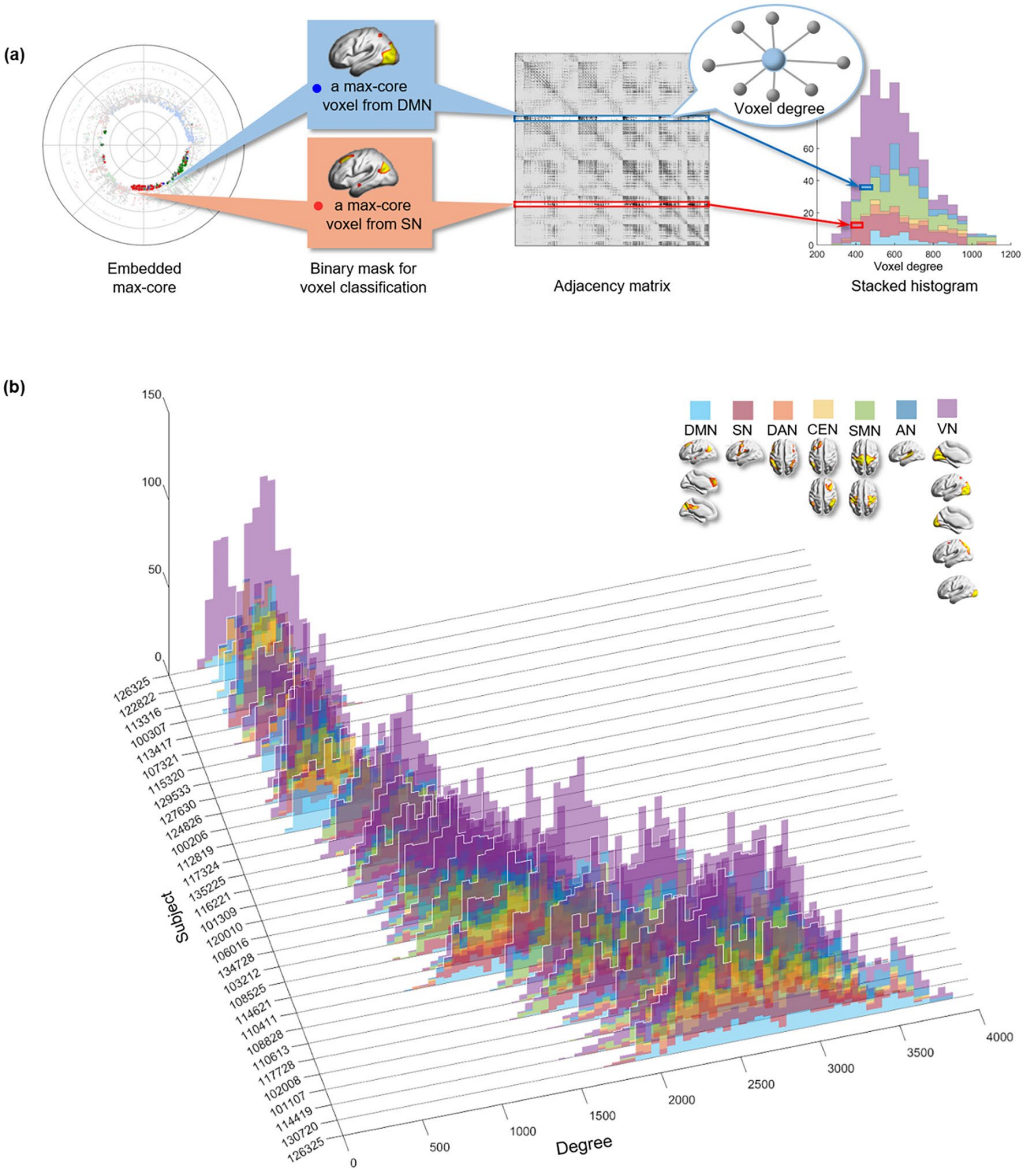
The stacked histogram of the degree distribution of *k*_max_-core voxels calculated from the adjacency matrix used as input. (**a**) After finding the *k*_max_-core of a subject with *k*-core percolation, we read the degree of each *k*_max_-core voxel on the adjacency matrix. We classified *k*_max_-core voxels into seven categorical independent components (ICs) and produced a stacked histogram showing each *k*_max_-core voxel’s affiliation and the voxel degree simultaneously. (**b**) We depicted *k*_max_-core of 30 subjects. The voxel degrees of *k*_max_-core voxels and their affiliation are in different colors. *k*_max_-core voxels located on the right side of the histogram were deemed to have a greater degree initially in the adjacency matrix, indicating that they have connections with non-*k*_max_-core voxels as well as within *k*_max_-core voxels. In contrast, *k*_max_-core voxels on the left side of the histogram denote a relatively smaller voxel degree, implying that it has fewer connections with non-*k*_max_-core voxels and is thus almost confined to obtain connections with *k*_max_-core voxels. The histograms of 30 subjects were sorted in ascending order with the mean degree of the *k*_max_-core voxels in 3-dimensional space.

We grossly found three patterns of *k*_max_-core voxel-IC composition among individuals using functional labels: DMN-dominant, VN-dominant, and uneven but distributed (Fig. 7, Supplementary Fig. 11). When a dominant IC (DMN or VN) occupies more than 40% of *k*_max_-core voxels, the individuals were deemed to be DMN-dominant or VN-dominant (Figs. 1, 7, Supplementary Figs. 11a,b, 12a,b). An individual without any dominant IC and showing distributed (*k*_max_-core) voxel-IC compositions was named as having a distributed pattern (Figs. 1, 7c, Supplementary Figs. 11c, 12c). There were five individuals with a DMN-dominant pattern, 18 individuals with a VN-dominant pattern, and seven individuals who had a distributed pattern (Supplementary Fig. 11). The *k*_max_-core voxel sizes of the individuals with the distributed pattern were significantly greater than those of the individuals with VN- or DMN-dominant patterns (*p* < 0.05). Stacked histograms of the degree distribution of *k*_max_-core voxels are presented in Fig. 8, and it should be noted that all 30 individuals, regardless of the degree maximum of each individual, could be annotated with one of the three types of *k*_max_-core voxel-IC compositions. An exemplary case showed the changes of k-cores in terms of IC-voxel rendering on brain template along k-core percolation (Supplementary Fig. 13).

## Discussion

In this study, we applied hyperbolic embedding and *k*-core percolation to investigate the latent topology of the human functional brain network. Hyperbolic embedding provides insightful visualization of the hierarchical like nature of the functional brain network, and k-core percolation discovers the core structure of individuals. The coordinates of voxels by hyperbolic embedding enable us to measure the functional within-themselves proximity of specific subnetworks of the brain. VNs and the parietal lobe showed functional proximity at rest frequently in individuals. The *k*-core percolation disclosed which subnetworks contributed to forming core voxels at maximum and that there were individual variations in dominant (visual or DMN) or distributed patterns on the *k*_max_-core voxels-ICs composition analysis.

Previous studies reported hierarchical organization of human brain network. Centrality measure is one of the most commonly used methods to investigate hierarchical nature of human brain^4^. Node degree and betweenness centrality are well-known measures; however, they only show a local or global property^5^. Node degree measures local influence since they include only neighbor nodes in calculation. The betweenness centrality of a node is calculated based on shortest path length, which considers whole network. However, k-core percolation provides hierarchy in their structures considering both the local and the global connections at each step of k-core decomposition^41^ along with dynamic re-checking the degree within k-core of the networks while removing the nodes of k-1 shell^27–29^. The nodes of outer k-shells are locally central, while ones in the central shells are globally central. Hence, the k-core percolation reveals topological importance of the network. It peels the network layer by layer to reveal the most internal one and provides hierarchical fingerprints^29^. In this study, we aimed to discover one unique central core which has the densest connection and pivotal hub of individual’s brain network.

The *k*-core percolation has recently been interpreted as one part of explosive percolation^42^, which was once considered to be discontinuous but later continuous^43^ and finally proposed to be hybrid^44^ in its configuration dynamics according to the percolation process. In explosive percolation in complex networks, dynamic changes in network configuration were observed/simulated in a forward way, meaning how global connectivity was formed with the addition of new edges to the networks. The formation of the largest component could have been discontinuous, continuous or hybrid^42–44^. In contrast, optimal percolation was also introduced to find the vulnerable nodes for targeted attack and trial of dismantling^11,12^ eventually to have defined influencer nodes^10^. This idea has become more popular for understanding epidemic spread, power grid failure^14,15^ and social message propagation^13,25^, either viral or fake, and has introduced algorithms of collective influence or belief propagation^13^. Optimal percolation tried to reveal a minimal set of nodes and thus kept itself different from *k*-core percolation decomposition, saying that *k*-core percolation finds a group of nodes not pinpointing the nodes with the highest collective influencer score (for a message, electrical power, epidemic spreading capability). In the optimal percolation or collective influence algorithm, of course, they proceed backward from the largest component to fragments. We followed the idea of *k*-core percolation; the largest component with the initial input and the adjacent matrix was reconfigured while removing the *k*-shell in this investigation.

Either forward or backward, regardless of the name and the use of specific percolation in the application context, for investigation of complex brain networks, we need to identify the voxels of highest interest and their grouping to a certain category, i.e., functional or anatomical label. When we have a sufficient number of voxels, such as more than 5000 in this study, we do not need to find each voxel for its contribution to maintaining resting-state brain function but want to find the groups of voxels of interest that remain after parsimonious filtration, in other words, percolation. *k*-core percolation functioned as the tool to find the survivors of this endeavor. Adoption of the *k*-core percolation algorithm from the literature^27–29,45^ easily yielded the voxels of *k*_max_-core. We applied k-core percolation to each participant’s brain network which consisted of about 5000 voxels to reveal the innermost central core of the hierarchical structure. After reaching *k*_max_, if one goes a step further (by incrementing the value of *k* by one), the largest component was disintegrated into many smaller pieces. As expected, the degree distribution of voxels belonging to the ICs of these *k*_max_-cores ranged from the highest to the mid-level (Supplementary Figs. 9, 10).

In the resting state, fMRI renders information on the fluctuation of BOLD signals per voxel, and evidently, the individuals are conscious, although sometimes their minds are drifting from introspection/imagination to paying attention to the milieu, full of MRI radiofrequency-derived noise or the scenes within the gantry. Individual differences in this mindset are expected, and temporal fluctuations will add up to make the stationary setting. In this study, we assumed perfect stationarity, meaning we calculated a single-digit correlation between every voxel *i* and voxel *j*, which led us to define 25 million or more possible edges. After scrutinizing the correlation, which ranged from − 0.7 to 0.8 in most cases, we wanted the binarized networks to be scale-free. That a network is a scale-free network means that the structure of the network has a similar structure independent of the scale of observation. In detail, most nodes have a few connections^31–34^, while some nodes have larger number of functional connections with other nodes. The scale-freeness, heterogeneity of degree distribution or power law obeyed by degree PDF on log–log plots is reflected by the innate topological features of the functional brain network, such as the hierarchical organization of nodes by their edges^5^. We cut off the edges with the threshold value of 0.4, so that each network meets scale-free criteria while retaining most of nodes in the single largest component. This yielded approximately 300,000 to 3,900,000 edges, with more than 80% of voxels remaining among 5937 resized voxels. Only the positive intervoxel correlation was included in the analysis in this study, and the negative correlation (anticorrelation) and its contribution to the multilayered duplex interdependent brain network remain to be studied. Such is also the case with the temporal variation in *k*_max_-core voxels and their IC-voxel composition.

Using the adjacency matrix and its consequent network, which defined the stationary voxel correlations at rest on rsfMRI, we proceeded to visualize the configuration in 2 dimensions with the hyperbolic disc. All the voxels were designated to belong to one (rarely more than one) IC, and the behavior of dynamic change of surviving voxels at each step of *k*-core percolation was presented using flag plots (Figs. 3c, 4c,d) and on the embedded hyperbolic discs (Fig. 4a, Supplementary Fig. 5a,b). Flag plots of voxels belonging to seven large representative ICs are displayed in Supplementary Fig. 7. It was interesting that the size of voxels varied among ICs before *k*-core percolation; however, *k*-core percolation allowed certain IC voxels, such as SMN, auditory network (AN), or central executive network (CEN), to vanish. Another point of merit is that voxels belonging to the SN disappear completely in a few individuals but remain in the others. Regarding DAN, CEN, AN, voxels seemed to vanish, but a small number of voxels belonging to these ICs remained definitely and joined the group of voxels of *k*_max_-core. This interesting phenomenon on *k*-core percolation is just reported here and will be the subject of further study to understand the conscious resting-state of mind in normal individuals and its correlates on rsfMRI.

Conscious individuals, evidently in an awake resting state on examinations, and their electrophysiological or perfusion correlates on electroencephalography (EEG), magnetoencephalography (MEG) or rsfMRI were studied by various investigators to yield a representative theory of consciousness, such as global neuronal workplace theory and integrated information theory (IIT)^46–51^. Global neuronal workplace theory^47,50^ advocated distributed subnetworks interconnected onto each other in conscious states. Thus, the isolated subnetworks are not the correlates for consciousness, and instead, once connected in a network of subnetworks, consciousness is achieved, emphasizing input–output processing. In contrast, integrated information theory^48,49^ measures cause-effect power with maximally irreducible integrated information in some areas, most likely suggesting the posterior area of the brain. Irrespective of which theory suits the data better, the subnetworks participating in the maintenance of consciousness should be discovered on each modality (EEG, MEG, or rsfMRI). If it is true that the posterior area contributes to the conscious resting state, as indicated by IIT, we need to investigate whether the VN we found in this study would be in charge. A perturbation study and/or calculation of integrated information, ϕ, of IIT will also be necessary^51^. The method we introduced in this study will be a good platform for visualization of intervoxel correlations and for elucidation of the changes in IC-voxel composition upon *k*-core percolation using a flag plot for these studies.

Discovery that the *k*_max_-core voxels of the parietooccipital area are dominant in more than half of the studied individuals and that VNs obviously participate in the *k*_max_-core in the remaining distributed or DMN-dominant individuals would mean that posterior or parietooccipital areas are one of the important correlates of resting-state consciousness. This indicates that everyone had VN in their *k*_max_-core voxels (Supplementary Figs. 7, 10). Interestingly, in the individuals with *k*_max_-core voxels uneven-but-distributed belonging to all the ICs, the contribution as core structure by all the other ICs seemed equivalent. The meaning of this phenomenon might be understood by looking into the temporal fluctuation of *k*_max_-core voxel-IC investigations in future studies.

Referring to the high angular coherence on the hyperbolic disc embedding and the dominance for *k*_max_-core on *k*-core percolation, the VNs had the strongest connectivity within IC, and their dominance in *k*_max_-core voxels was significant in half of the individuals at rest. The VN has been reported as one of the major hub regions in previous studies^52^. The VN is a unimodal area that conducts highly specialized functions; for example, the primary visual cortex, well delineated by cytoarchitectonic features, is an important correlate of corresponding functional vision. In contrast, the domain-general frontal area, involved in various cognitive tasks^53^, showed low functional proximity within itself. The VN also has a higher neuronal density than the others. Considering that the resting functional connectomes are not engaged at any activation tasks, the unimodal highly differentiated subnetwork might be the one with strength in angular coherence and contribution to the coreness on *k*-core percolation. In the same vein, a sensorimotor network also showed high angular coherence. However, it was not found to be dominant in any individual but just one of the components weakly or absent contributors to the *k*_max_ core (Supplementary Fig. 7).

The voxels belonging to the precuneus network remained in the *k*_max_ core in most of the individuals. The precuneus is an associative region that is especially involved in self-related information processing^54,55^. The precuneus is also a crucial component of the DMN, which was also found to be a dominant IC subnetwork in a fraction of the individuals in this study, reminding us that the DMN is a well-known subnetwork active in the resting state. Although the precuneus is not inherently highly differentiated and specialized like a visual system, in the resting state, the voxels of the precuneus came to join the *k*_max_ core.

The core of the individuals showed diversity in size and IC voxel composition. This IC-voxel composition pattern was arbitrarily classified among the individuals: DMN-dominant, VN-dominant, and uneven but distributed types. Individuals with DMN or VN dominance revealed that their *k*_max_-core voxels consisted of (1) mainly DMN and several minor ICs or (2) VN and CEN and few minors in the resting state, respectively. This might imply that dominant IC subnetworks are sustained as characteristics of the individuals and/or that in every individual, there is a fluctuation of mental states, which drift between the distributed pattern and, in some instances, enter the DMN major or the VN major states, which would also be observed by rsfMRI. Former or latter, whichever interpretation might be the fact/truth, further study with sliding window segmentation and phase-shift observation of rsfMRI is warranted.

Individuals with the distributed pattern tended to have a larger size of voxels in their *k*_max_-core, and more than half of all had voxels belonging to VNs or DMN in their *k*_max_-core. It is interesting to know whether DMN- or VN-dominant patterns might be a drifting accentuation of these two subnetworks, while evenly distributed IC voxels are the background default state at rest in humans. Then, DMN and VN-dominant patterns are the two extremes of the spectrum, and the distributed pattern resides between them. Regarding hubs, there was a controversy that in some reports^56,57^, hub regions were composed of voxels belonging to various subnetworks and not confined to a specific system in one report. In other reports^58^, there was a predominance of the visual system and precuneus among hub nodes. We suggest that this *k*_*max*_ core IC-voxel composition be used as a fingerprint to identify and describe an individual’s physiological or pathological characteristics of their resting IC compositions of the cores.

Finally, finding *k*_*max*_ core voxels using an established visualization method on hyperbolically embedded discs accompanying *k*-core percolation raised the possibility of studying stationary and dynamic functional connectivity of voxels and their hierarchy upon filtration/percolation. The *k*-core percolation disintegrated the initial largest component gradually but sometimes abruptly; thus, this descent pattern seemed to represent the core and subcore configuration of voxels hidden under just the simple-looking scale-free functional brain network. The hidden relation between areas/regions/voxels on rsfMRI was recently investigated with either a coactivation pattern (CAP)^59^ or hidden Markov model (HMM)^60,61^, both of which followed the success of elucidation of dynamic changes of various CAPs on the analysis of MEG data^62^ or of discovering variable HMM states and finding the transition between HMM states at rest using MEG^63^, respectively. On the temporal scale and/or cross-modal investigations, both CAP and HMM methods were used to understand the twitches and other trivial movement/activities of humans during imaging^64^ and eventually consciousness. Our method of *k*_max_-core detection and the annotation of the *k*_max_-core voxels to ICs upon filtration will lead us to define the hierarchical structure of core-periphery coherent gathering of voxels. The 2-dimensional display of hyperbolic discs allowed us to visualize how the *k*_max_-core was formed by the simple rule of *k*-shell peeling or decomposition. This is the new platform to understand finally the awake, twitching intermittently, mentally drifting with various attention to milieu or his/her mind in the MRI gantry in a conscious resting-state of human individuals. Temporal variation^65^ with rsfMRI and cross-modal investigation with MEG or EEG^66^ will be the next step of investigation using the current method of hyperbolic disc embedding and *k*-core percolation.

### Limitations

There are some limitations to our approach. There is no gold standard for selecting threshold on functional brain network. To investigate the hierarchical structure of the functional network using hyperbolic embedding and k-core percolation, we thresholded networks to meet scale-freeness, which means we assumed the functional brain network is scale-free^67^. It implies self-similarity and heterogeneity of degree distribution. To test the results of k_max_-core depending on thresholding, we selected one representative participant of each three pattern (VN/DMN/Distributed pattern) and applied various thresholds (0.3, 0.4, and 0.5). Despite the arbitrariness of threshold, patterns of k_max_-core voxels were consistent (Supplementary Figs. 14–16). Although we used two criteria for thresholding; scale-freeness and node inclusion criteria, the optimal thresholding criteria based on quantitative estimation is needed to provide in this approach.

This study only included positive correlation and investigated static brain network. The negative correlation is considered the other side of the coin, and the model including both positive and negative correlation is needed^68^. Dynamic change of brain network is also extensively investigated and suggested continuous shifting in neural correlates^60,61,69,70^. Further studies using both positive, negative correlation and for dynamics of functional connectivity hierarchy are warranted.

## Methods

### Dataset

We included 180 participants from the Human Connectome Project (HCP) S1200 release, which is available with open access (www.humanconnectomeproject.org/study/hcp-young-adult/data-use-terms). This open access data is available to those who register an account at ConnectomeDB and agree to the open access data use terms. The acquisition parameters and preprocessing steps were described in Ref.^71^. All methods were carried out in accordance with relevant guidelines and regulations. Participants were free of neurological diseases and psychiatric disorders (31 participants with age range 22–25 years, 84 participants with age range 26–30 years, and 64 participants with age range 31–36 years; male: 76, female: 104). We included all 180 subjects in angular coherence analysis and 30 subjects in *k*-core percolation analysis (ten participants with age range 22–25 years, ten participants with age range 26–30 years, and ten participants with age range 31–36 years; male: 15, female: 15).

### Preprocessing of the rsfMRI data

rsfMRI was obtained with a 3 T scanner with the following parameters: TR = 720 ms; TE = 33.1 ms; flip angle = 52°; FOV = 208 × 180 mm, 2 mm isotropic voxels. The minimally preprocessed data from HCP were further preprocessed using Statistical Parametric Mapping (SPM, www.fil.ion.ucl.ac.uk/spm/) and FMRIB Software Library (FSL, fsl.fmrib.ox.ac.uk/fsl/)^71^. EPI images were corrected gradient and motion-induced distortion, and a field map-based nonlinear transform was also used to correct distortion. After images were coregistered and normalized into standard space, intensity normalization was performed. These minimal preprocessing results in 2 × 2 × 2 mm sized voxel images^71^. We additionally conducted smoothing using 6 mm full-width at half maximum (FWHM) of the Gaussian kernel, and bandpass filtering (0.01–0.1 Hz). Finally, we downsampled the data to reduce the computational load of voxel-based whole-brain network analysis (dimension: 31 × 37 × 31, voxel size: 6 × 6 × 6 mm^2^), and 5937 voxels that consisted of the gray matter were used.

### Functional/anatomical label of voxels

We performed independent component analysis (ICA) using MELODIC (multivariate exploratory linear optimized decomposition into independent components) to extract resting-state networks^36^. Fifteen independent components were classified after manual inspection of spatial maps: aDMN, DMN, PCN (equivalent to posterior DMN), SN, DAN, left CEN (L CEN), right CEN (R CEN), SMN1, SMN2, AN, VN1, VN2, VN3, VN4, and VAN. The spatial maps were manually inspected and classified based on previous studies^72,73^. To present our results in a more comprehensible summary, we also combined 15 resting-state networks into seven categories: DMN, SN, DAN, CEN, SMN, AN, and VN. Both schemes were used for functional label. The Brainnetome atlas was used to generate 15 left/right anatomical lobes, and seven lobes consisted of bilateral brain regions for anatomical labeling^40^.

### Assessment of functional connectivity and voxel composition of subnetworks

For the scale of the 5937 cubic isotropic voxels, we measured blood oxygen level-dependent (BOLD) signals from each voxel of fMRI data and characterized the spontaneous fluctuations over the time series, ${\upsigma }^{2}\left(X\right)$, where X is the time series of the BOLD signal. For the BOLD-fMRI time-series X = (X_1_…, X_N_) of a given voxel, the variance was computed by the sample variance ${\widehat{\upsigma }}^{2}\left(X\right)$ given as the following formula:

1 $${\widehat{\upsigma }}^{2}\left(X\right) =\frac{1}{N-1}{\sum }_{i=1}^{N}{\left({X}_{i}-\overline{X }\right)}^{2},$$

where $\overline{X }$ denoted the sample mean of $X$. Between a pair of voxels with BOLD-fMRI time-series X = (X_1_…, X_N_) and Y = (Y_1_…, Y_N_), the functional connectivity was estimated by the sample Pearson correlation coefficient $\widehat{\uprho }$:

2 $$\widehat{\uprho }\left(X,Y\right)=\frac{1}{N-1}{\sum }_{i=1}^{N}\frac{\left({X}_{i}-\overline{X }\right)\left({Y}_{i}-\overline{Y }\right)}{\widehat{\upsigma }\left(X\right)\widehat{\upsigma }\left(Y\right)}.$$

From the sample Pearson correlation coefficient $\widehat{\uprho }$ of each pair of voxels, we obtained a square matrix of Pearson correlation coefficients for each of the subjects. To determine the most appropriate threshold values for composing a binary network, we applied tentative threshold values for $\widehat{\uprho }$, followed by comparison of the degree distribution and the relative size of the largest connected component. We determined the threshold value, considering both the scale freeness of the degree distribution and the maximal inclusion of voxels as much as possible in the largest component. For scale freeness, we considered the degree distribution with a straight-formed line on a log–log scale plot as appropriate. For the size of the network, we considered the threshold low enough for the single largest component of a graph (which was embeddable on the disc) to contain 80% of the voxels in the brain. Consequently, we constructed an unweighted, undirected graph for each subject by applying the threshold to the correlation coefficient matrix.

### Hyperbolic disc embedding of networks into the ${\mathbb{S}}^{1}$/${\mathbb{H}}^{2}$ model

The resulting binary graph from each subject was mapped onto a hyperbolic disc using the ${\mathbb{S}}^{1} /{\mathbb{H}}^{2}$ geometric network model^31,34^. Connectomes were assumed to exist in underlying geometric space, linked with the observed topologic properties through a law of connection probability that defined the likelihood if the two regions of the brain were linked. In the model, the connection probability between two voxels $i$ and $j$ was determined by the hyperbolic distance between two voxels:

3 $${p}_{ij}=\frac{1}{1+{e}^{\frac{\upbeta }{2}\left({d}_{ij}-\widehat{R}\right)}},$$

where $\upbeta$ is the clustering coefficient of the network and $\widehat{R}$ is the outermost radial coordinate among the embedded voxels. The hyperbolic distance, ${d}_{ij}$, is usually determined by hyperbolic laws of cosines,

4 $$\text{cosh}\,k{d}_{ij} = \text{cosh}\,k{r}_{i}\,\text{cosh}\,k{r}_{j}\,\left(1-\text{tanh}\,k{r}_{i}\,\text{tanh}\,k{r}_{j}\,\text{cos}\Delta {\uptheta }_{ij}\right),$$

where* k* is the curvature of the plane, ${r}_{i}$ is the radial coordinate of the ith point and $\Delta {\uptheta }_{ij}$ is the angular separation between two points i and j, while

5 $${d}_{ij}\cong {r}_{i}+{r}_{j}+2\ln\frac{\Delta {\uptheta }_{ij}}{2},$$

was used as a good approximation, given that $k{r}_{i}$ and $k{r}_{j}$ are large enough and $\Delta {\uptheta }_{ij}$ that is not too small, for $\Delta {\uptheta }_{\text{ij}}>\sqrt{{\text{e}}^{-2{\text{r}}_{\text{i}}}+{\text{e}}^{-2{\text{r}}_{\text{j}}}}$) (This approximation is used for $\Delta {\uptheta }_{ij}$ that is not too small, for $\Delta {\uptheta }_{\text{ij}}>\sqrt{{\text{e}}^{-2{\text{r}}_{\text{i}}}+{\text{e}}^{-2{\text{r}}_{\text{j}}}}$). To determine the geometric object (i.e., a hyperbolic disc) that was most likely to generate the original binary graph, we used a software named *Mercator* (available at https://github.com/networkgeometry/mercator), introduced by García-Pérez et al.^31^, which applied maximum likelihood techniques and a machine learning approach for embedding the network. By this means, by computing the most appropriate set of polar coordinates for $N$ voxels $\left({\text{r}}_{1},{\uptheta }_{1}\right),\left({\text{r}}_{2},{\uptheta }_{2}\right),\dots \left({\text{r}}_{N},{\uptheta }_{N}\right)$ and the clustering coefficient $\upbeta$, we embedded the binary graph onto a hyperbolic disc for each subject.

### Angular coherence to assess the degree of gathering of subnetwork voxels on the hyperbolic discs

For the assessment of narrow or widespread aggregation of voxels in the hyperbolic disc space, we used a metric called angular coherence, which was previously devised to investigate how points were angularly similar on the hyperbolic disc^31^. The angular coherence $\upxi \in \left[\text{0,1}\right]$ of a set of points $X$ was determined as follows:

6 $$\upxi {\text{e}}^{{\text{i}}\upphi}=\frac{1}{\text{N}}{\sum }_{\text{k}\in X}{{\text{e}}^{{\text{i}} \uptheta_\text{k}}},$$

where ${\uptheta }_{\text{k}}$ is the coordinate of point $k$ and $\text{N}$ is the number of points (voxels in this study) in $X$. As intuitive from Eq. (6) The higher $\upxi$ was, the more points in the set were locally concentrated to form an angularly gathered structure. In this study, we used binarized (Z > 6) IC maps for functional labels and the Brainnetome atlas for anatomical labels. The degree of gathering of subnetworks on hyperbolic discs for resting-state voxel networks was estimated by calculating the angular coherences of the voxels included in the ICs or lobes.

### k-core percolation and composition of k-core and k_max_-core structures

We performed *k*-core percolation to investigate individual-specific core subnetworks of brain connectivity. The *k-core* of a network is the maximal subgraph of the network in which all vertices have a degree of at least $k$. The $k$-core was identified by removing all voxels with degrees less than $k$ and recalculating the degrees of all the remaining voxels until no voxel remained with a degree less than $k$. We used a pruning algorithm suggested by Azimi-Tafreshi et al.^29^. The voxels that belonged to the $k$-core but not to the $(\text{k}+1)$-core form the $k$-shell of the network, and they were said to have $k$-coreness. Along *k*-core percolation, the changes in the size of the subnetwork at the *k*-core were well visualized in these flag plots over increasing *k,* revealing the association between the coreness of voxels and subnetworks of ICs or lobes to which the voxels belonged. Voxels and their belonging to ICs or lobes were drawn as flag plots to describe how the number of voxels consisting of each IC/lobe decreased with increasing coreness *k*.

### Degree distribution of k_max_-core over functional/anatomical subsystems

As the coreness* k* increased, the step maximum was defined as the one where on step further, at ${k}_{max}+1$, all voxels constituting the largest component were disintegrated to smaller pieces. This phase transition always occurred abruptly and was thus discontinuous, making *k*_max_-core. We evaluated the size and degree distribution of this *k*_max_ core for ICs and lobes.

## Supplementary Information

Supplementary Information.

## Data Availability

The functional brain dataset in the present study is available for download on the Human Connectome Project (HCP) (https://www.humanconnectome.org/) with the acceptance of data use terms.
