# Supplementary material for: Characteristic functional cores revealed by hyperbolic disc embedding and k-core percolation on resting-state fMRI
Source: Sci Rep. 2022 Mar 22;12:4887. doi: 10.1038/s41598-022-08975-7 (PMC8941113; doi:10.1038/s41598-022-08975-7)
Supplement: Supplementary file 1 — Supplementary Information. [file 41598_2022_8975_MOESM1_ESM.docx]

***Supplementary information for***

**Characteristic functional cores revealed by hyperbolic disc embedding and *k*-core percolation on resting-state fMRI**

Wonseok Whi^1,2,3*^, Youngmin Huh^3*^, Seunggyun Ha^4^, Hyekyoung Lee^5^, Hyejin Kang^5#^, Dong Soo Lee^1,2,3#^

^1^Department of Molecular Medicine and Biopharmaceutical Sciences, Seoul National University

^2^Department of Nuclear Medicine, Seoul National University and Seoul National University Hospital

^3^Medical Research Center, Seoul National University

^4^Division of Nuclear Medicine, Department of Radiology, Seoul St. Mary's Hospital, College of Medicine, The Catholic University of Korea

^5^Biomedical Research Institute, Seoul National University Hospital

* Co-first authors, contributed equally

Corresponding authors

Dong Soo Lee M.D.Ph.D.

Seoul National University, Seoul Korea

dsl@snu.ac.kr 82-2-2072-2501

Hyejin Kang Ph.D.

Seoul National University Hospital, Seoul Korea

hkang211@snu.ac.kr 82-2-740-8542

**I. Supplementary tables**

**Supplementary Table 1. The average and standard deviation (SD) of angular coherence (AC) of ICs from repeated embedding.** In a subject (100206), hyperbolic disc embeddings were repeated 100 times, and ACs were calculated for IC subnetworks: default mode network (DMN), anterior DMN (aDMN), salience network (SN), dorsal attention network (DAN), left and right central executive network (L/R CEN), sensorimotor network 1/2 (SMN1/2), auditory network (AN), visual network 1/2/3/4 (V1/2/3/4), and visual attention network (VAN).

| IC | Mean | SD | CV |
| --- | --- | --- | --- |
| DMN | 0.72 | 0.06 | 0.08 |
| aDMN | 0.54 | 0.10 | 0.19 |
| PCN | 0.80 | 0.04 | 0.05 |
| SN | 0.62 | 0.01 | 0.02 |
| DAN | 0.33 | 0.12 | 0.36 |
| L CEN | 0.55 | 0.07 | 0.12 |
| R CEN | 0.57 | 0.01 | 0.02 |
| SMN1 | 0.78 | 0.05 | 0.06 |
| SMN2 | 0.62 | 0.01 | 0.01 |
| AN | 0.81 | 0.01 | 0.01 |
| VN1 | 0.84 | 0.02 | 0.02 |
| VN2 | 0.46 | 0.21 | 0.47 |
| VN3 | 0.53 | 0.08 | 0.15 |
| VN4 | 0.78 | 0.01 | 0.02 |
| VAN | 0.57 | 0.18 | 0.31 |

IC: independent component, SD: standard deviation, CV: coefficient of variation

**Supplementary Table 2. Demographic information of subjects.** In angular coherence analysis, 180 subjects were included. Thirty subjects were included in the *k*-core percolation analysis.

|  | | n=180 | n=30 |
| --- | --- | --- | --- |
| Age |  |  |  |
|  | 22-25 | 31 | 10 |
|  | 26-30 | 84 | 10 |
|  | 31-35 | 64 | 10 |
|  | 36+ | 1 | - |
| Gender |  |  |  |
|  | Male | 76 | 15 |
|  | Female | 104 | 15 |

**Supplementary Table 3. Angular coherence of each independent component (IC).** SD: standard deviation

| IC | Mean | SD | Median | Number of voxels |
| --- | --- | --- | --- | --- |
| DMN | 0.57 | 0.14 | 0.58 | 441 |
| aDMN | 0.64 | 0.18 | 0.66 | 158 |
| PCN | 0.48 | 0.15 | 0.50 | 224 |
| SN | 0.65 | 0.17 | 0.69 | 351 |
| DAN | 0.50 | 0.18 | 0.51 | 363 |
| L CEN | 0.75 | 0.16 | 0.79 | 285 |
| R CEN | 0.66 | 0.15 | 0.69 | 428 |
| SMN | 0.81 | 0.15 | 0.86 | 294 |
| SMN | 0.75 | 0.13 | 0.78 | 267 |
| AN | 0.63 | 0.16 | 0.66 | 289 |
| VN1 | 0.71 | 0.15 | 0.74 | 286 |
| VN2 | 0.72 | 0.15 | 0.76 | 443 |
| VN3 | 0.80 | 0.18 | 0.86 | 253 |
| VN4 | 0.75 | 0.22 | 0.84 | 205 |
| VAN | 0.56 | 0.16 | 0.58 | 321 |

**Supplementary Table 4. Angular coherence of each lobe.** SD: standard deviation

| Lobes | Mean | SD | Median | Number of voxels |
| --- | --- | --- | --- | --- |
| L Frontal | 0.29 | 0.13 | 0.29 | 830 |
| R Frontal | 0.30 | 0.13 | 0.30 | 820 |
| L Temporal | 0.29 | 0.12 | 0.28 | 521 |
| R Temporal | 0.25 | 0.12 | 0.23 | 495 |
| L Parietal | 0.25 | 0.12 | 0.24 | 526 |
| R Parietal | 0.26 | 0.13 | 0.24 | 549 |
| L Insula | 0.57 | 0.20 | 0.57 | 58 |
| R Insula | 0.62 | 0.19 | 0.62 | 58 |
| L Limbic | 0.40 | 0.15 | 0.39 | 115 |
| R Limbic | 0.40 | 0.17 | 0.40 | 86 |
| L Occipital | 0.71 | 0.19 | 0.76 | 320 |
| R Occipital | 0.72 | 0.19 | 0.78 | 327 |
| L Subcortical | 0.54 | 0.23 | 0.55 | 212 |
| R subcortical | 0.48 | 0.21 | 0.47 | 207 |
| Cerebellum | 0.36 | 0.18 | 0.35 | 813 |

**Supplementary Table 5. The mean numbers of *k*_max_-core voxels of 30 subjects were calculated using functional labels.** Fifteen resting-state independent component (IC) networks were used. SD: standard deviation

| IC | Mean | SD | Total voxels  belonging to IC |
| --- | --- | --- | --- |
| DMN | 78 | 73 | 441 |
| aDMN | 10 | 13 | 158 |
| PCN | 50 | 32 | 224 |
| SN | 83 | 71 | 351 |
| DAN | 48 | 56 | 363 |
| L CEN | 18 | 25 | 285 |
| R CEN | 36 | 36 | 428 |
| SMN1 | 91 | 95 | 294 |
| SMN2 | 85 | 77 | 267 |
| AN | 80 | 72 | 289 |
| VN1 | 118 | 81 | 286 |
| VN2 | 140 | 100 | 443 |
| VN3 | 100 | 70 | 253 |
| VN4 | 35 | 36 | 205 |
| VAN | 106 | 74 | 321 |

**Supplementary Table 6. The mean numbers of *k*_max_-core voxels of 30 subjects were calculated using functional labels.** Seven categories combining 15 independent components (ICs) were used. SD: standard deviation

| IC | Mean | SD | Total voxels  belonging to categorized IC |
| --- | --- | --- | --- |
| DMN | 120 | 90 | 732 |
| SN | 83 | 71 | 351 |
| DAN | 48 | 56 | 363 |
| CEN | 52 | 53 | 682 |
| SMN | 151 | 141 | 483 |
| AN | 80 | 72 | 290 |
| VN | 354 | 228 | 1104 |

**Supplementary Table 7. The mean numbers of *k*_max_-core voxels of 30 subjects for 15 anatomical labels.** SD: standard deviation

| Lobes | Mean | SD | Total voxels  belonging to lobe |
| --- | --- | --- | --- |
| R Frontal | 77 | 60 | 830 |
| L Frontal | 76 | 58 | 820 |
| R Temporal | 54 | 41 | 521 |
| L Temporal | 59 | 45 | 495 |
| R Parietal | 125 | 86 | 526 |
| L Parietal | 152 | 97 | 549 |
| R Insula | 6 | 8 | 58 |
| L Insula | 7 | 9 | 58 |
| R Limbic | 13 | 13 | 115 |
| L Limbic | 9 | 10 | 86 |
| R Occipital | 109 | 72 | 320 |
| L Occipital | 109 | 76 | 327 |
| R Subcortical | 2 | 4 | 212 |
| L Subcortical | 2 | 4 | 207 |
| Cerebellum | 26 | 30 | 813 |

**Supplementary Table 8. The mean numbers of *k*_max_-core voxels of 30 subjects for categorized anatomical labels.** SD: standard deviation

| Lobes | Mean | SD | Total voxels  belonging to each bilateral lobes |
| --- | --- | --- | --- |
| Frontal | 153 | 117 | 1650 |
| Temporal | 113 | 84 | 1016 |
| Parietal | 277 | 180 | 1075 |
| Insula | 12 | 17 | 116 |
| Limbic | 23 | 23 | 201 |
| Occipital | 218 | 146 | 647 |
| Subcortical | 4 | 7 | 419 |
| Cerebellum | 26 | 30 | 813 |

**II. Supplementary figures**


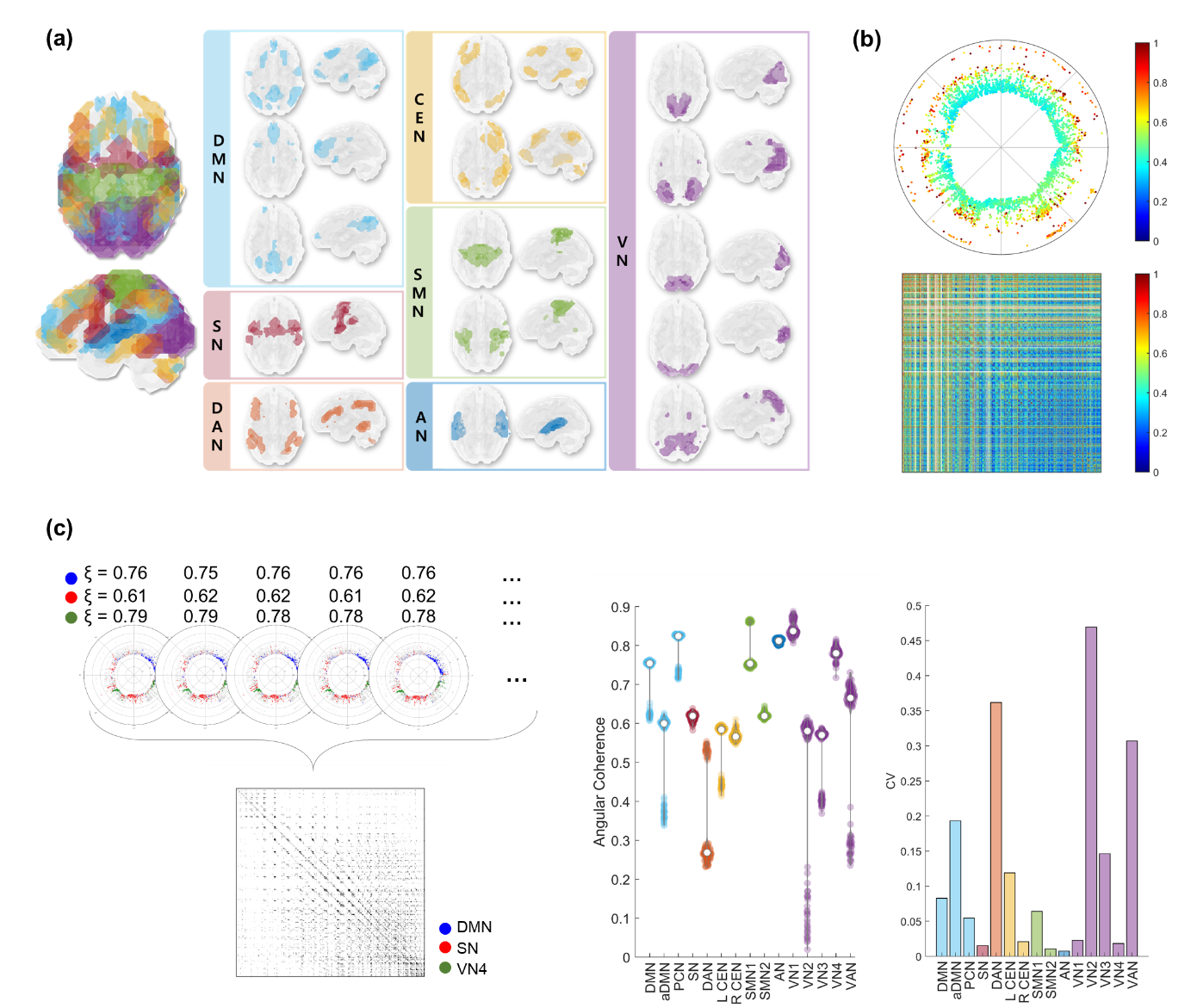


**Supplementary Figure 1. Summary of the methods: hyperbolic disc embedding and its reproducibility and *k*-core percolation with rendering upon the hyperbolically embedded discs.** (a) Fifteen independent components (ICs) derived from the independent component analysis were rendered in the 3D brain. The default mode network (DMN), anterior DMN (aDMN), and precuneus network (PCN) are printed in pale blue, the salience network (SN) is printed in red, and the dorsal attention network (DAN) is printed in orange. The left/right central executive network (L/R CEN) is shown in yellow, sensorimotor networks 1/2 (SMN) in green, auditory network (AN) in navy blue, and visual networks (V1/2/3/4/VAN) in violet. All ICs were shown in both axial and sagittal views. (b) The reproducibility of hyperbolic embedding is shown for one example individual (subject #100206) with 100 repetitions. Using this repeatedly embedded 5,937 voxels 100 times, the intervoxel distances in the hyperbolic disc were considered edges. After thresholding, voxels not belonging to the largest component and edges not found valid were shown in white in the matrix. The coefficient of variation (CV) of intervoxel distances is shown as a matrix (bottom). For a voxel, all the CVs of distances of its edges connecting with all the other valid edges were averaged to yield its representative CV of embedding reproducibility. On one embedded disc, CVs per voxel are depicted with colors of the jet color map. CVs per voxel ranged from 0.29 to higher values, the voxels near the center were found to be put (embedded) reproducibly, and the voxels outside to the periphery of the disc showed higher variability of the intervoxel distances with all the other voxels (their locations on the repeated embedding moved around the axis angle and thus the distance). (c) We also calculated the angular coherence (AC) of each embedding using 15 ICs (left), and the distribution of AC for each IC is displayed (right). The CV of each IC was shown in a bar plot (right). Notably, the reproducibility of AC over repeated hyperbolic disc embedding shows the reproducibility of the IC-voxel configuration on the embedded disc despite rotation/reflection symmetry and branch permutation symmetry of the hyperbolic disc owing to its geometric characteristics.


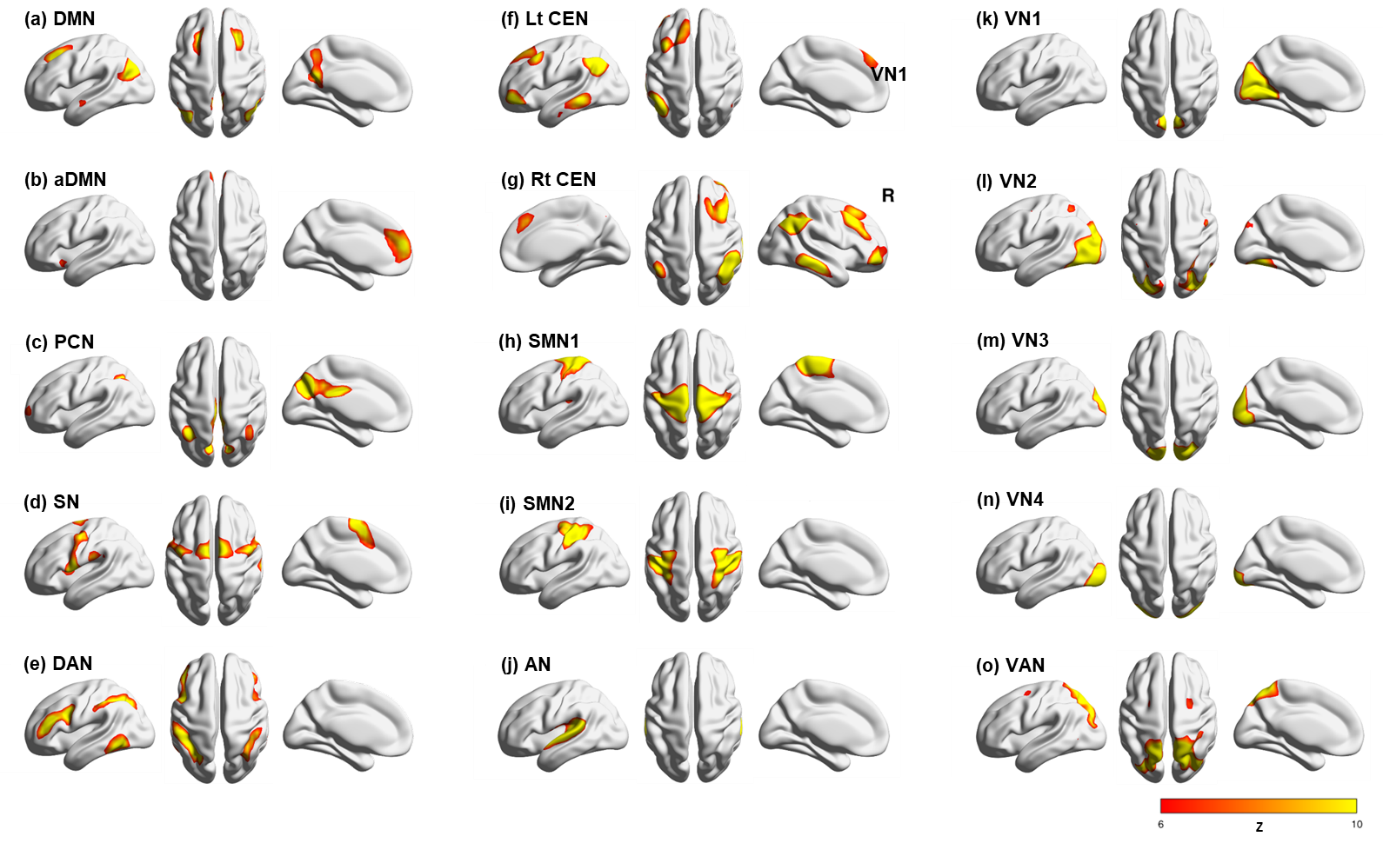


**Supplementary Figure 2. Fifteen independent component (IC) subnetworks and their functional labels.** 180 individuals’ rsfMRI data were preprocessed and resampled into 6x6x6 mm^3^ and were put into group independent component analysis. The spatial maps of ICs were illustrated on the brain surface (Z > 6). (a) Default mode network (DMN), (b) anterior DMN, (c) precuneus network (PCN), (d) salience network (SN), (e) dorsal attention network (DAN), (f) left central executive network (L CEN), (g) right CEN (R CEN), (h) sensorimotor network 1 (SMN1), (i) SMN 2, (j) auditory network (AN), (k) visual network 1 (VN1), (l) VN2, (m) VN3, (n) VN4, (o) visual attention network (VAN).


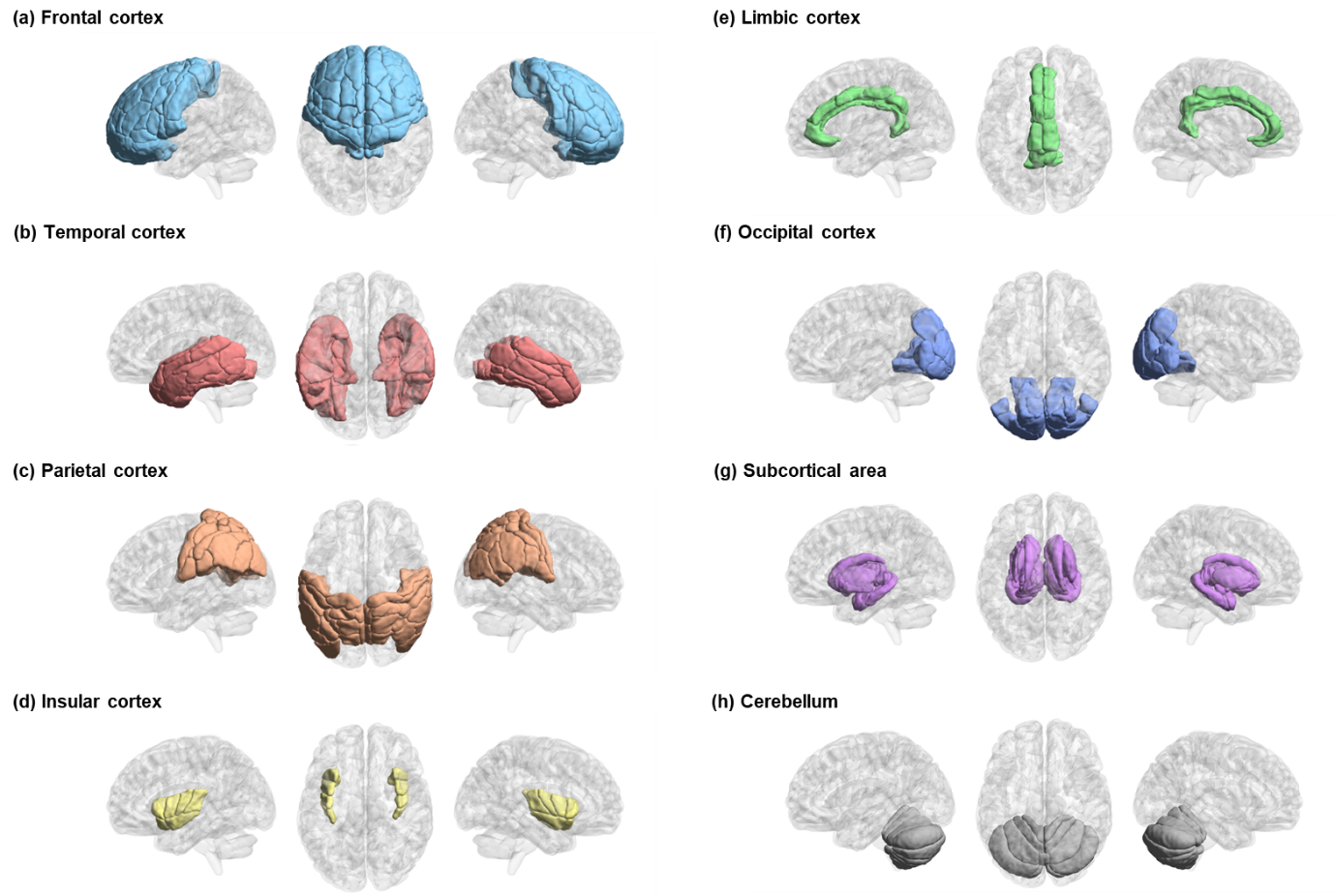


**Supplementary Figure 3. Anatomical labels and their voxels rendered on the 3-dimensional brain.** Eight brain lobes parcellated based on the Brainnetome atlas: (a) bilateral frontal lobes, (b) bilateral temporal lobes, (c) bilateral parietal lobes, (d) bilateral insular cortex, (e) bilateral limbic cortex, (f) bilateral occipital lobes, (g) bilateral subcortical area, and (h) cerebellum. Considering the right and left lobes for the first seven and the cerebellum (containing vermis), fifteen anatomical labels were used for analysis.


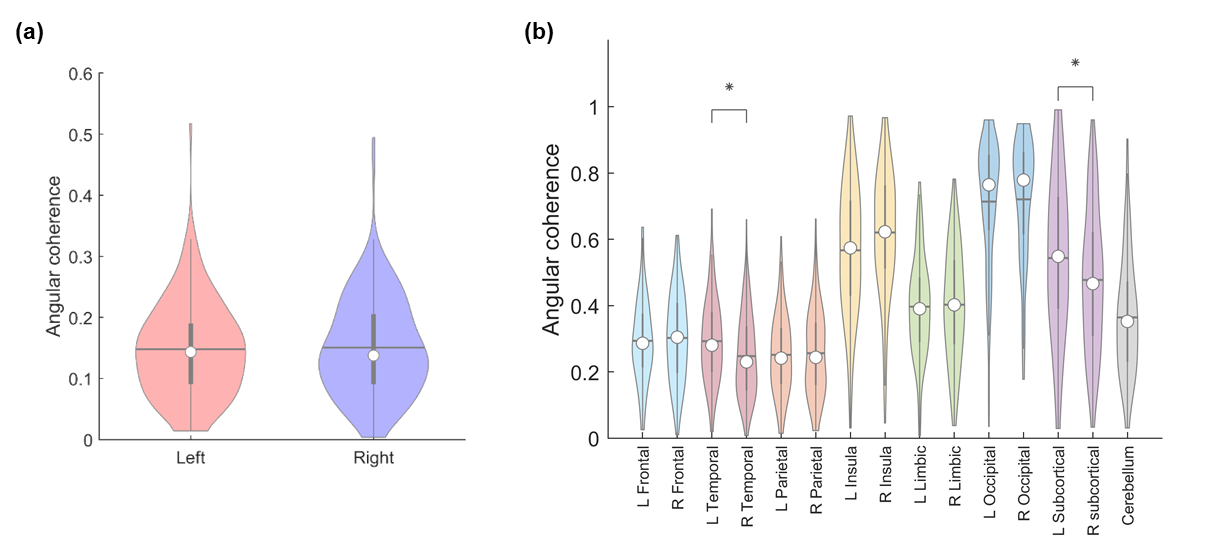


**Supplementary Figure 4. The comparison of angular coherence between the hemispheric voxels or lobe voxels of the left and right hemispheres.** Whether there is any hemispheric laterality in angular coherence was investigated. (a) Designating every voxel into the left or right hemisphere, there was no significant difference. (b) Differences between the seven lobes on the left and right: frontal, temporal, parietal, insula, limbic, occipital lobe, and subcortical region. The temporal lobes and subcortical regions showed significant differences between the left and right (*p* < 0.05, FWER corrected). The left temporal lobe and left subcortical region showed significantly greater angular coherence than the right temporal lobe and left subcortical region. No difference in angular coherence in the other left and right lobes was found.


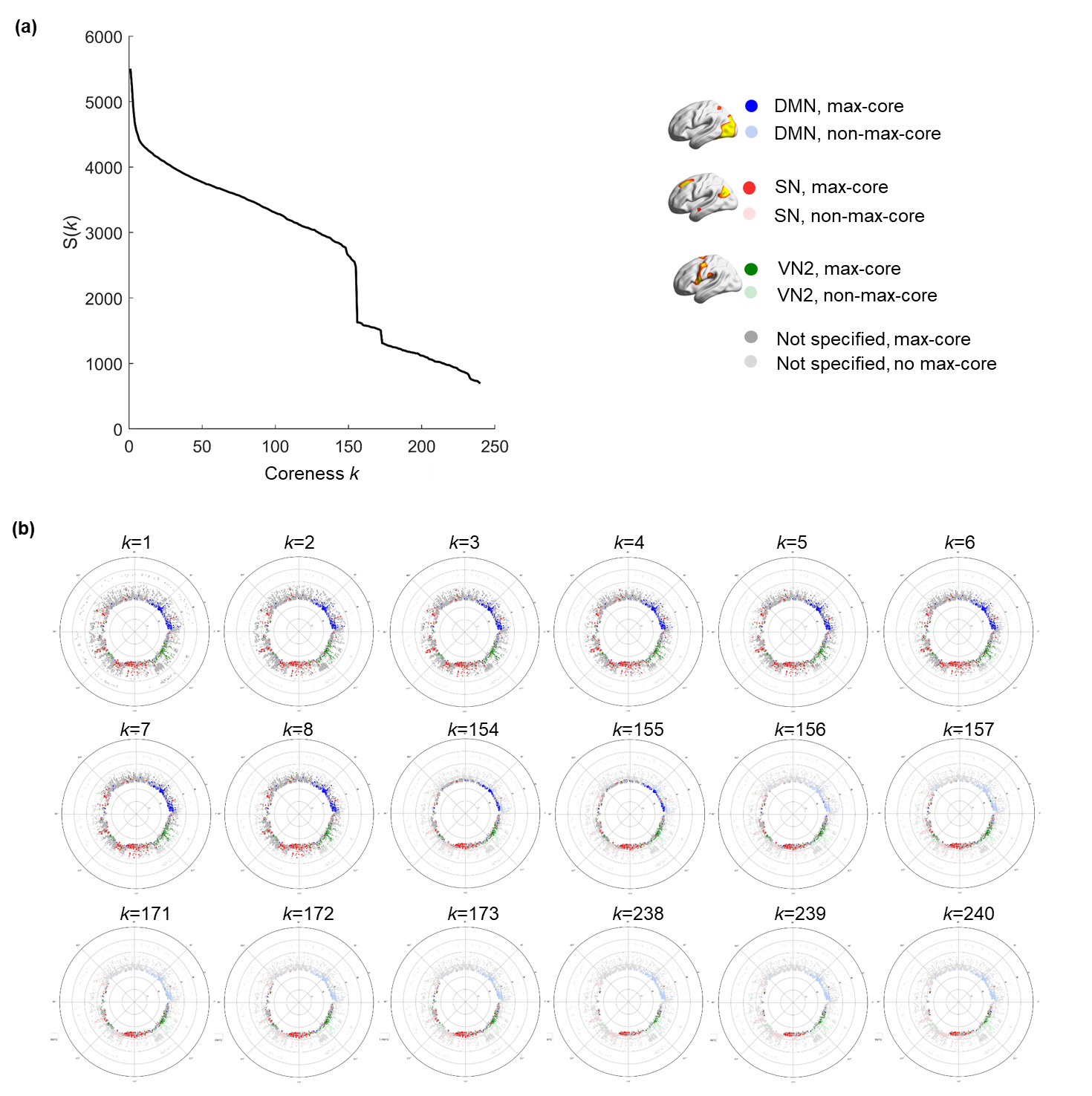


**Supplementary Figure 5. *k*-cores derived from the stepwise *k*-core percolation procedure on the hyperbolically embedded discs.** One can find the changing *k*-core subnetworks of an individual at the sampled *k*-core steps. The *k*-core percolation algorithm removes voxels with *k*-degrees, and the algorithm iterates by incrementing coreness *k* by 1. The set of voxels left after each *k*-core percolation step is called the *k*-core. The elimination procedure entails recalculating the degrees of remaining voxels, and the procedure stops when the largest component is fragmented into pieces and the largest component is no longer designated. In an example case (subject #100206), (a) The plot shows the size of the *k*-core, S(*k*), according to the coreness *k*. Both gradual and abrupt decreases are shown. (b) The *k*-cores that showed abrupt decreases (*k*=1, 2, 3, 4, 5, 6, 7, 8, 154, 155, 156, 157, 171, 172, 173, 238, 239, 240) were embedded on the hyperbolic discs. Each voxel that belongs to the default mode network (DMN), salience network (SN), and visual network (VN) 2 is colored blue, red, and green circles, respectively. The gray circles denote voxels that do not belong to the DMN, SN, and VN2. The voxels not included in the *k*-cores are shown in pale gray. As *k* increases, more voxels near the edge are eliminated, and an abrupt decrease in S(*k*) involves the mass desertion of voxels from *k*-cores. Even the voxels with higher degrees near the center were removed near the end of *k*-core percolation (examples; *k*=156, 172).


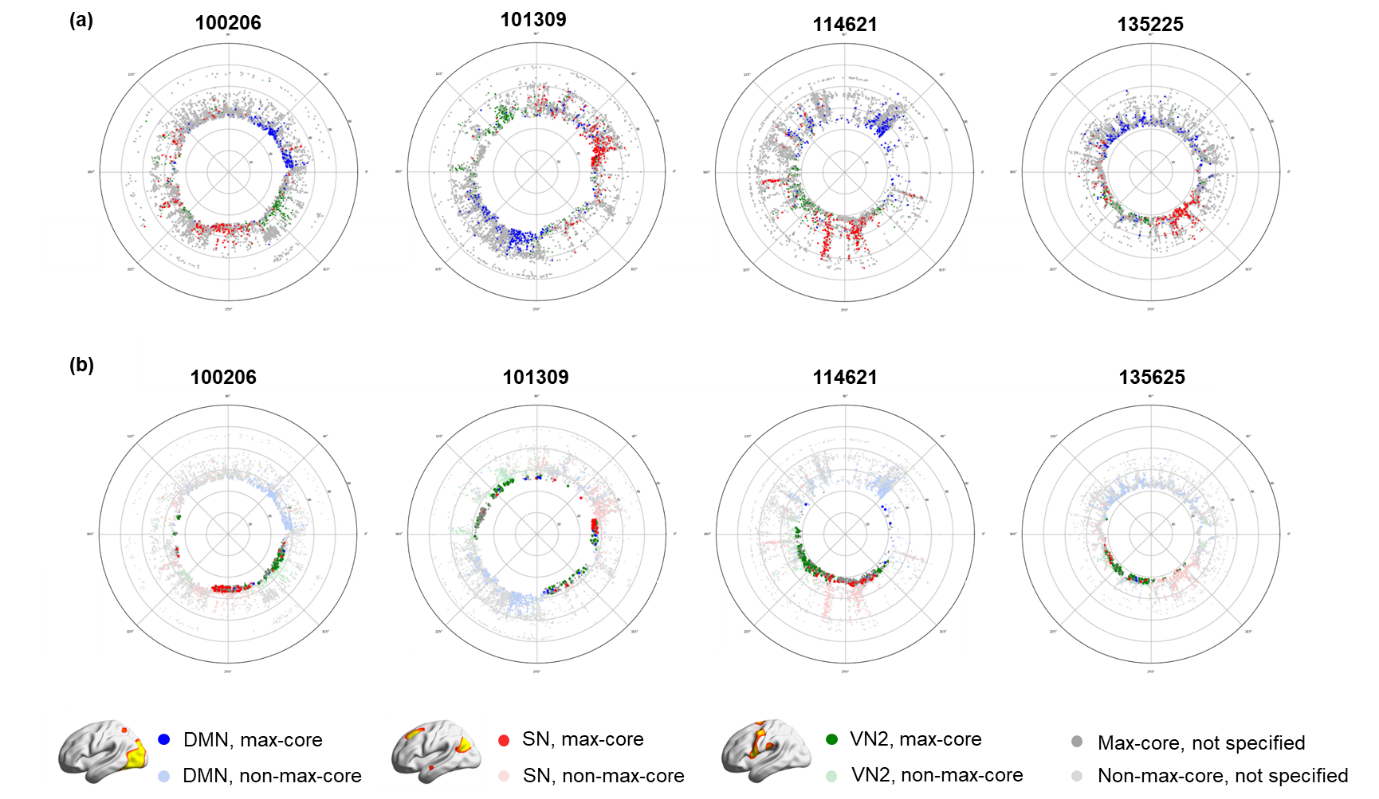


**Supplementary Figure 6. The *k*-cores (*k* = 1 and max) of four individuals** (subjects #100206, #101309, #114621, and #135225) **were embedded on their own hyperbolic discs.** (a) Representative voxels on the hyperbolic discs. Voxels that belong to the default mode network (DMN), salience network (SN), and visual network 2 (VN2) are shown as blue, red, and green circles, respectively. The gray circle represents voxels that belong to none of the three. Individuals showed similar but arbitrarily unique patterns of hyperbolic embedding of voxels: a larger circle (#100206, #101309), a circle with an outer radial pattern (#114621), and a smaller circle (#135225). Voxels from each network also show various patterns. The SN voxels of an individual (#100206) are distributed in a broad area, whereas those of another individual (#101309) form a cluster. The other (#114621) shows a radial pattern of a cluster consisting of SN voxels. (b) The *k*_max_-core voxels from *k*-core percolation in the above four subjects were visualized on the hyperbolic discs. Voxels that do not belong to *k*_max_-core are shown in pale. The numbers of *k*_max_-core voxels and to which independent components they belong vary widely between individuals. Each individual’s ID was shown on top of the disc.


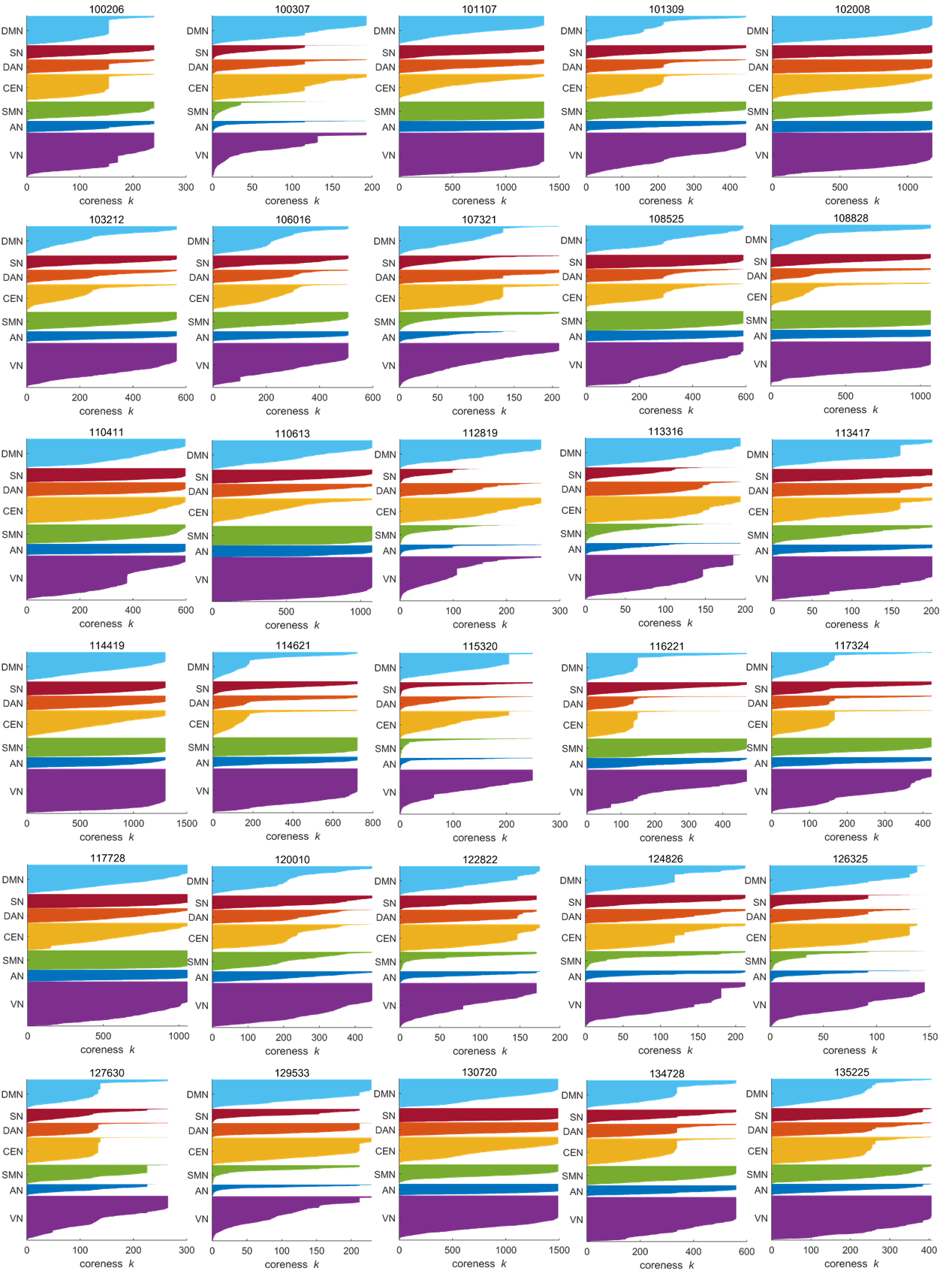


**Supplementary Figure 7. Flag plots of changes in independent component (IC)-voxel composition along *k*-core percolation.** The *k*-core percolation peels off the layer of the brain network of voxels having degree *k*. As the coreness *k* increases, the size of the *k*-core decreases. The flag plot shows the change in *k*-cores of each individual according to the coreness *k*. Every voxel that belongs to each IC is shown on the y-axis, and the horizontal bar of each voxel reaches the rightmost until the maximum coreness k. Categorical functional labels were used to visualize the default mode network (DMN), salience network (SN), dorsal attention network (DAN), central executive network (CEN), sensorimotor network (SMN), auditory network (AN), and visual network (VN). The voxels of an IC are sorted in descending order of the voxel’s *k* within each flag. Since there are voxels that belong to multiple ICs, the number of voxels in the y-axis is slightly greater than 5,937, the total number of voxels. Every individual shows unique patterns along *k*-core percolation: 1) abrupt or gradual decrease in the size of *k*-core voxels belonging to each IC along *k*-core percolation and 2) changes in the proportions of voxels that belong to seven categorical ICs.


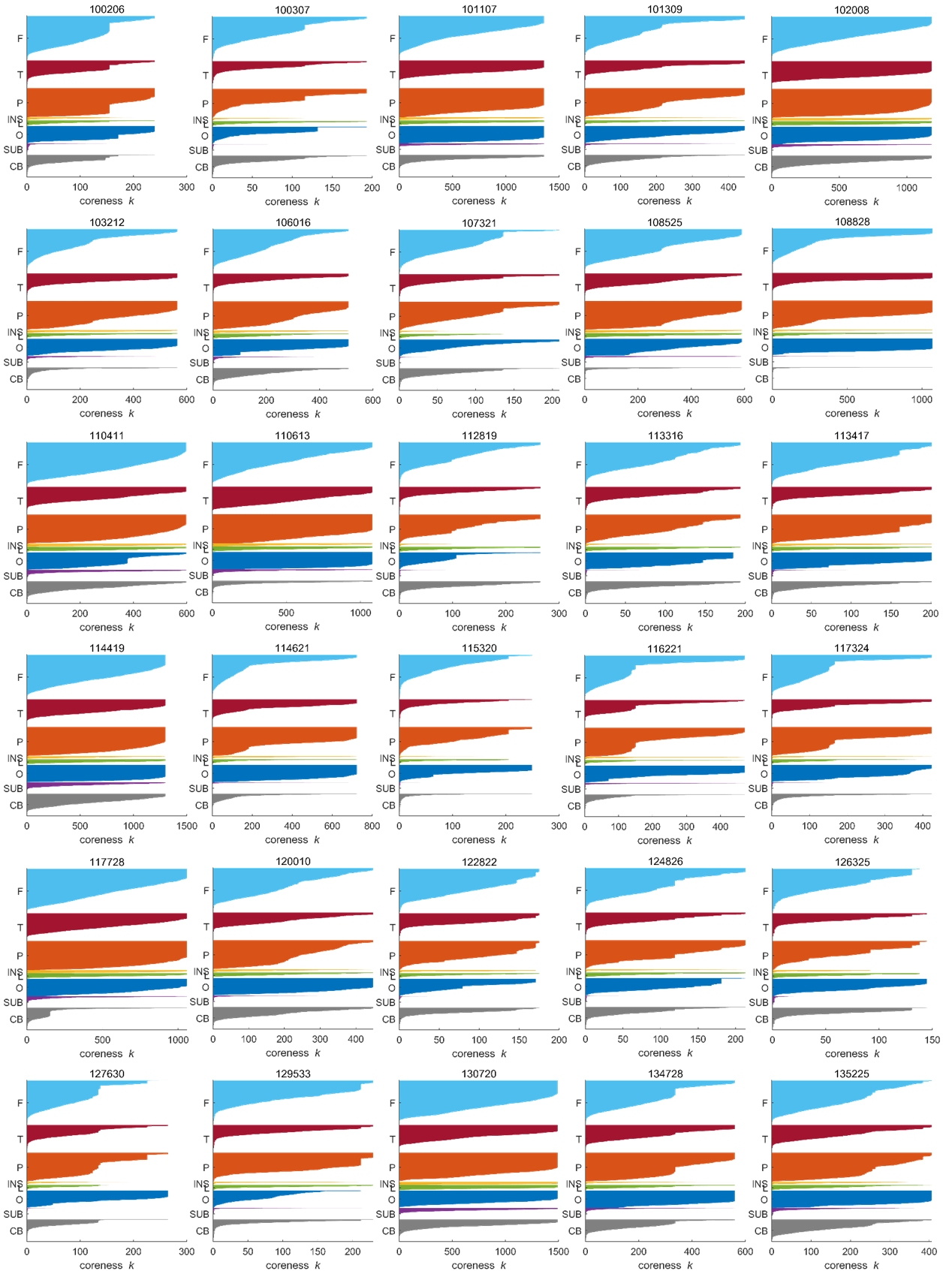


**Supplementary Figure 8. Flag plots of changes in lobe-voxel composition along *k*-core percolation.** Categorical anatomical labels were used to annotate the voxels into eight lobes: frontal lobe, temporal lobe, parietal lobe, insula, limbic system, occipital lobe, subcortical region, and cerebellum. Each lobe includes voxels from both the left and right hemispheres. The 5,937 voxels were labeled on the y-axis, and the horizontal bar of each voxel reached the rightmost position until the maximum coreness *k*. The voxels from each lobe were sorted in descending order for the numbers of lobe voxels.


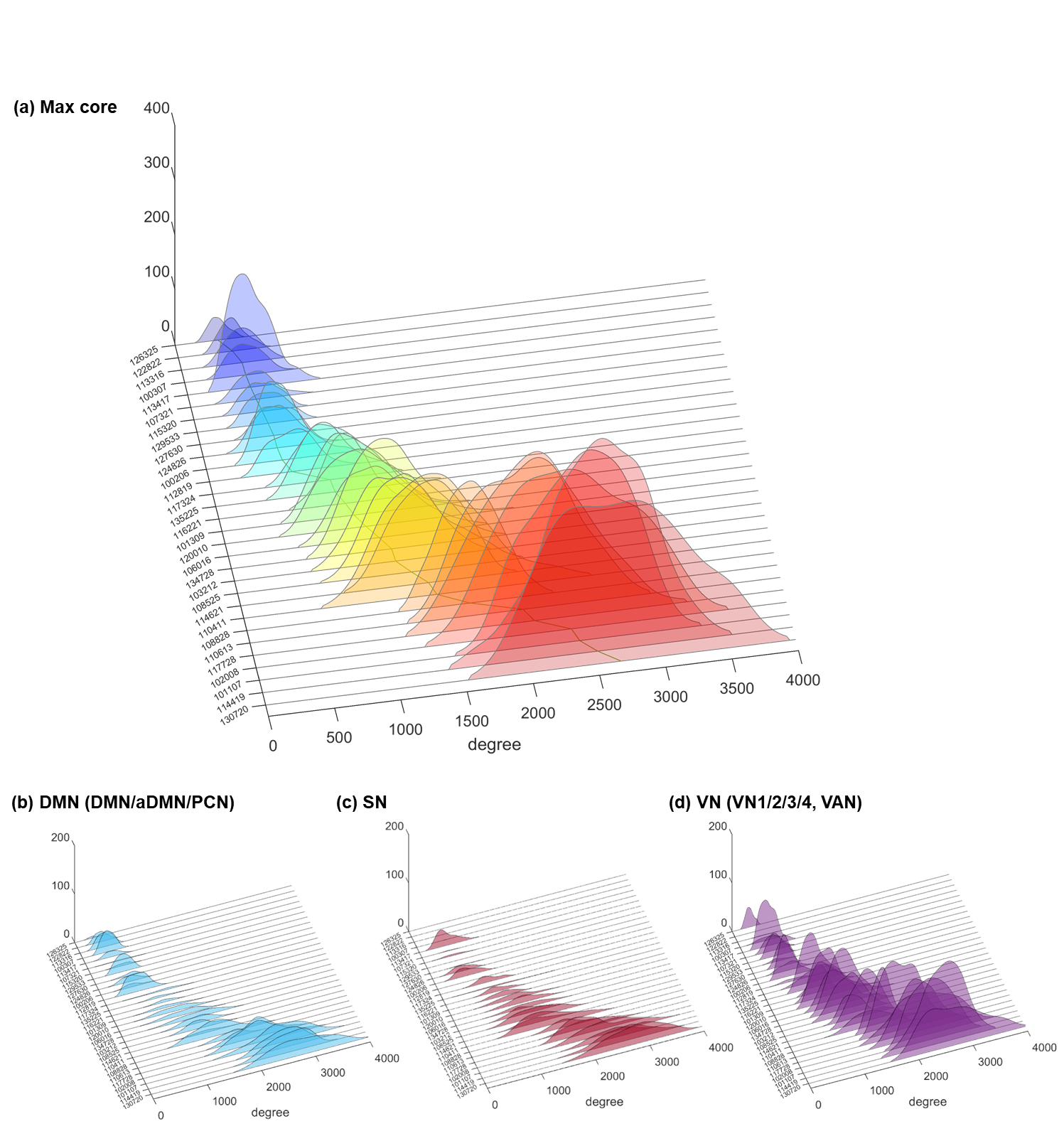


**Supplementary Figure 9. The histogram of *k*_max_-core voxels showing their degree distribution.** The degrees of *k*-core voxels were adopted from their initial adjacency matrix. (a) The histograms of degree distribution were visualized in different colors for individuals. The histograms were sorted in ascending order with the mean degree of the *k*_max_-core voxels. An individual at the top with a deep blue histogram has the lowest voxel degrees, and another with a red at the bottom has the highest. The functional label was used to annotate voxels. (b) The degrees of *k*_max_-core voxels belonging to the categorical default mode network (DMN; DMN/anterior DMN (aDMN)/precuneus network (PCN)), (c) salience network (SN), and (d) visual network (VN; VN1/2/3/4, visual attention network (VAN)) were displayed similarly to (a). The degrees of *k*_max_-core voxels include not only the voxels with dense connections with the colleague *k*_max_-core voxels but also the voxels with lower degrees but are effectively connected preferentially with *k*_max_-core connections. In optimal percolation, they called these voxels influencer nodes.


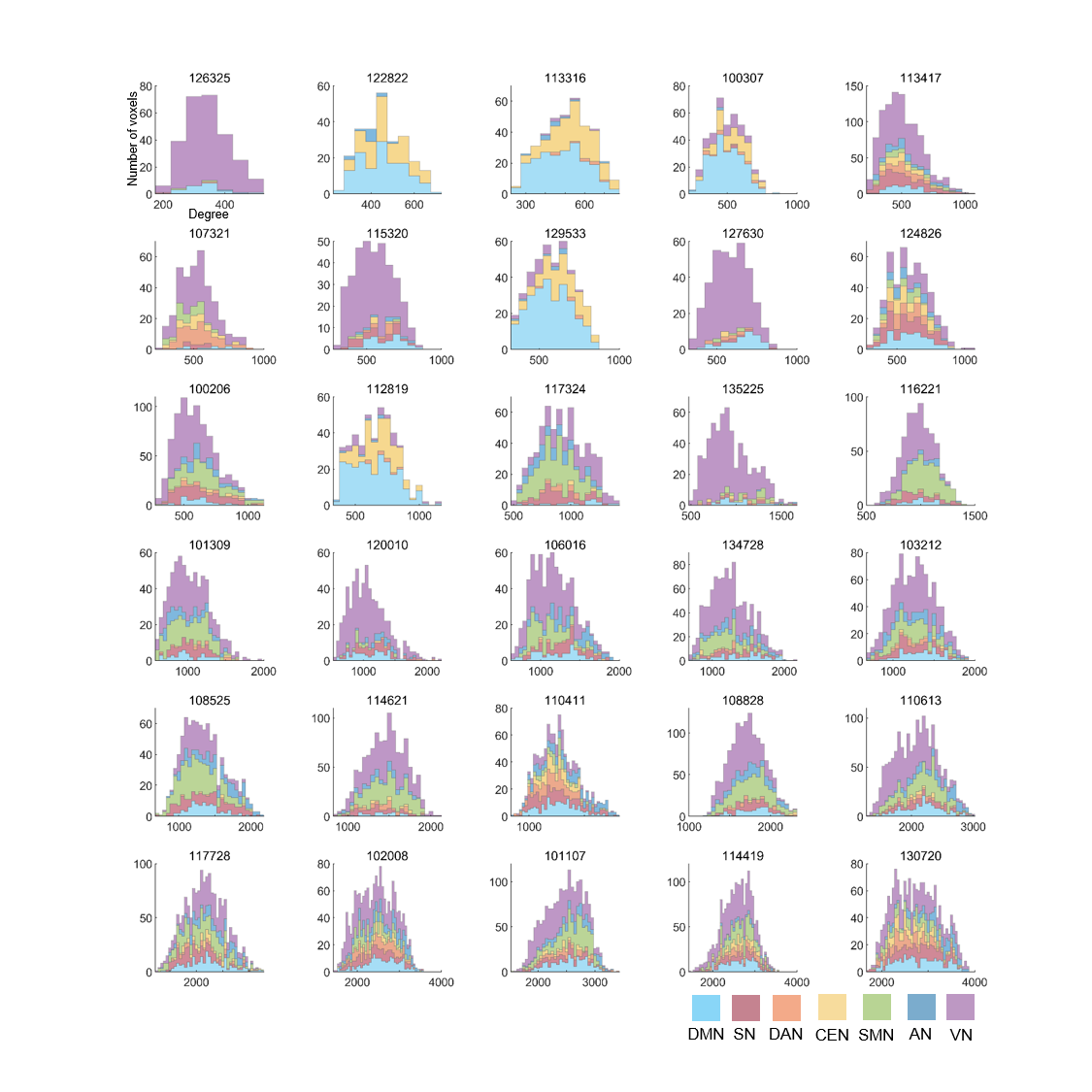


**Supplementary Figure 10. The stacked histogram of the degrees of *k*_max_-core voxels per individual.** The voxel degree was derived from the adjacency matrix, and the affiliation of each voxel is presented in different colors. A *k*_max_-core voxel located on the rightmost side of the histogram indicates that the voxel has the greatest degree in the adjacency matrix, indicating that it has many connections with non-*k*_max_-core voxels. In contrast, another *k*_max_-core voxel from the left to the rightmost denotes a relatively smaller degree. However, it belongs to *k*_max_-core, implying that it has connections mainly with other *k*_max_-core voxels, and only the surplus of degrees is used for connecting itself with non-*k*_max_-core voxels. The histograms of 30 subjects were displayed one by one with sorting in ascending order to the mean degrees of the *k*_max_-core voxels.


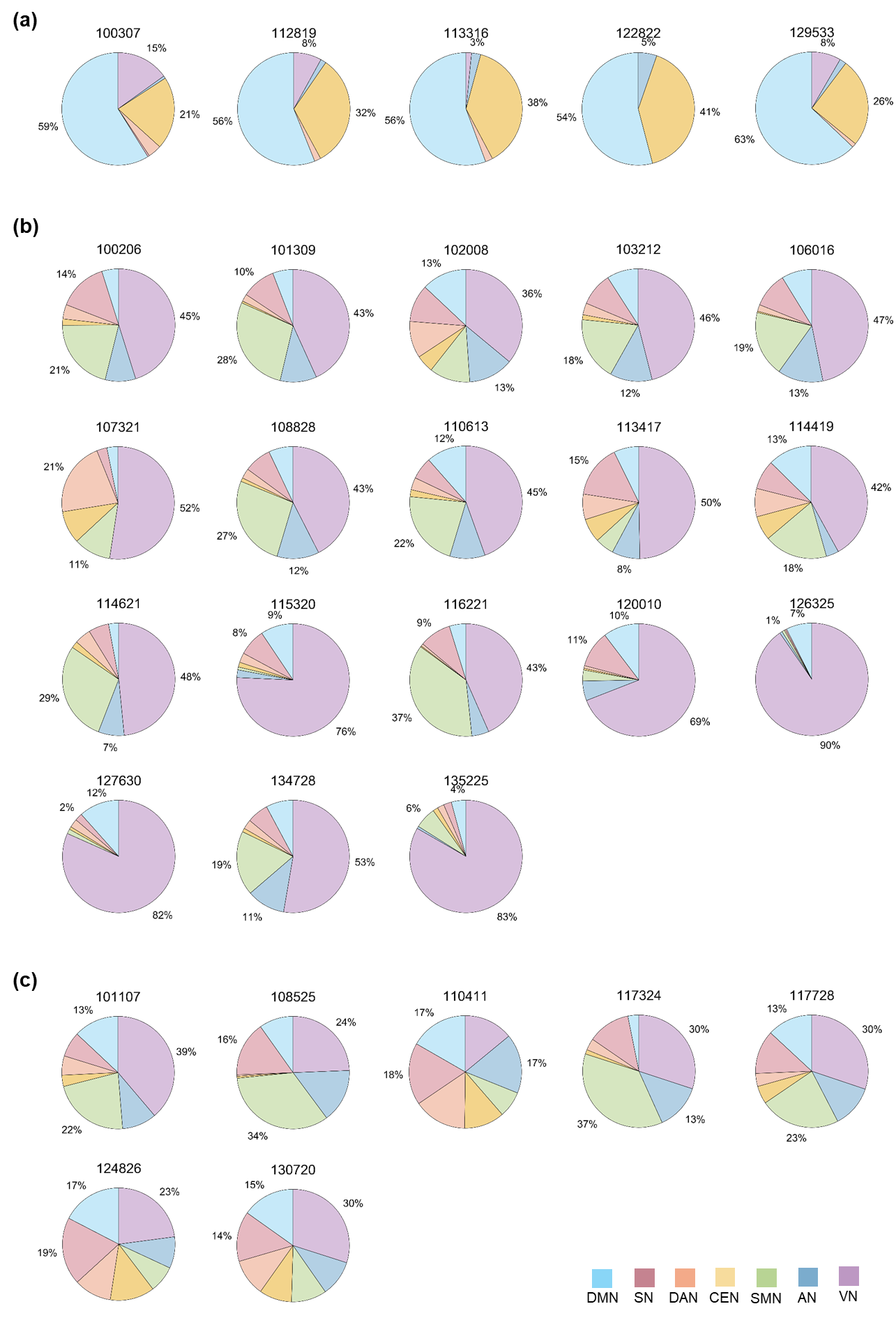


**Supplementary Figure 11. The pie plots show the ratio of *k*_max_-core voxels of each independent component (IC) over all *k*_max_-core voxels.** We used a categorical functional label that includes the default mode network (DMN), salience network (SN), dorsal attention network (DAN), central executive network (CEN), sensorimotor network (SMN), auditory network (AN), and visual network (VN). The affiliation of *k*_max_-core voxels of each individual is shown in the pie plot, and percentages of the three IC voxels with the greatest sizes are written around the plot. The individuals were assigned into three patterns by the IC-voxel composition of *k*_max_-core voxels. (a) An individual was grouped as DMN-dominant if more than 40% of *k*_max_-core voxels belonged to the DMN. (b) In a VN-dominant, more than 40% of the *k*_max_-core voxels belonged to the VN. (c) A distributed pattern indicates that there is no dominant IC for *k*_max_-core voxels. We found five DMN-dominant subjects, 18 VN-dominant subjects, and seven subjects with the distributed pattern.


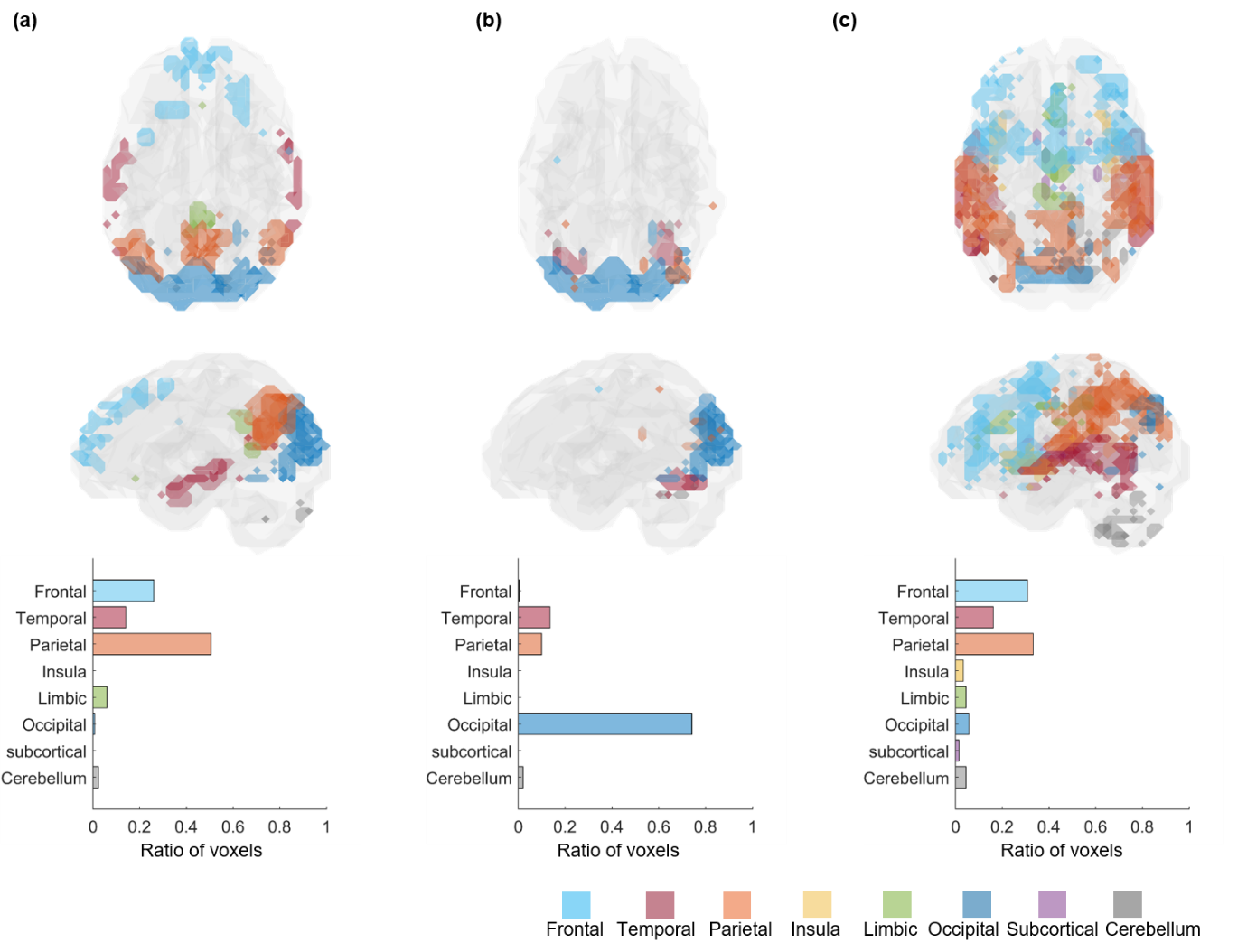


**Supplementary Figure 12. The *k*_max_-core of three individuals from Fig 7 is shown using the anatomical label.** We performed *k*-core percolation to find each individual’s *k*_max_-core voxels and affiliated these *k*_max_-core voxels with the lobes. Categorized anatomical labels, including the frontal lobe, temporal lobe, parietal lobe, insula, limbic system, occipital lobe, subcortical region, and cerebellum, were used to annotate *k*_max_-core voxels. Three patterns of *k*_max_-core voxel-IC composition were found in the three individuals, as shown in Fig 7. (a) In the first individual (129533), *k*_max_-core voxels were mostly in the parietal, temporal, and frontal lobes. (b) In the second individual (126325), *k*_max_-core voxels were mostly in the occipital lobe and a few in the temporal and parietal lobes. (c) In the third individual (110411), *k*_max_-core voxels were distributed in the frontal, temporal, and parietal lobes and in the other lobes. The ratios of the *k*_max_-core voxels of each lobe over the total number are shown as bar plots at the bottom.
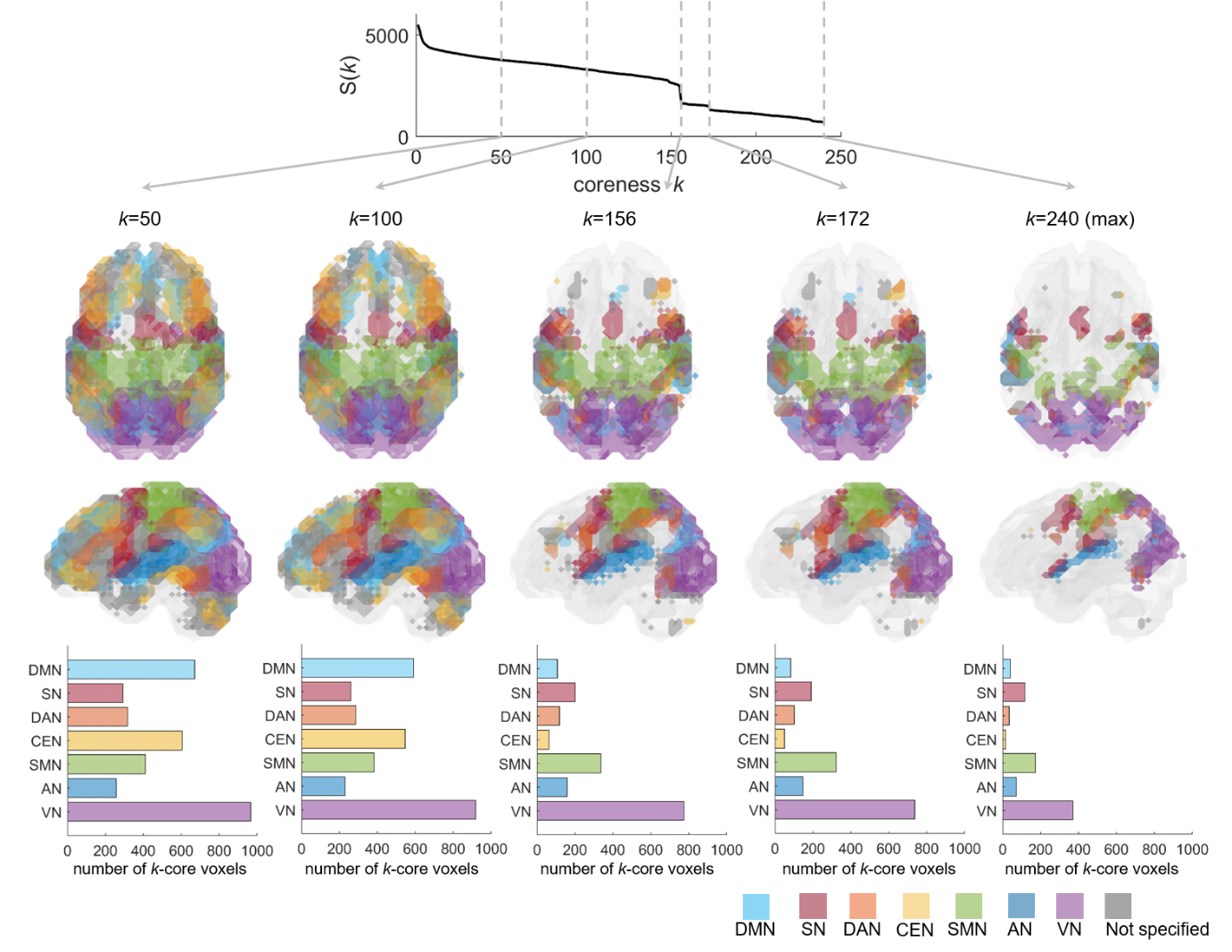


**Supplementary Figure 13. *k*-cores of an individual according to the coreness *k* from *k*-core percolation.** The plot on the top shows the size of the *k*-core, S(*k*), according to the coreness *k,* while *k*-core percolation proceeds of this individual (100206). We used a categorical functional label, which includes seven combined independent components (ICs), to classify *k*_max_-core voxels. Each voxel is printed on the 3-dimensional brain in corresponding colors in the middle. The bar plots on the bottom show the number of *k*_max_-core voxels that belong to each IC. As coreness *k* increases, the number of voxels belonging to each IC decreases, and at the step maximum, *k*_max_-core voxels remain. Voxels belonging to the visual network (VN) are more than 900 when *k* is 1 and come to be less than 400 at the *k*_max_ step. However, VN occupies the majority since the number of *k*_max_-core voxels belonging to other ICs comes to be far fewer.


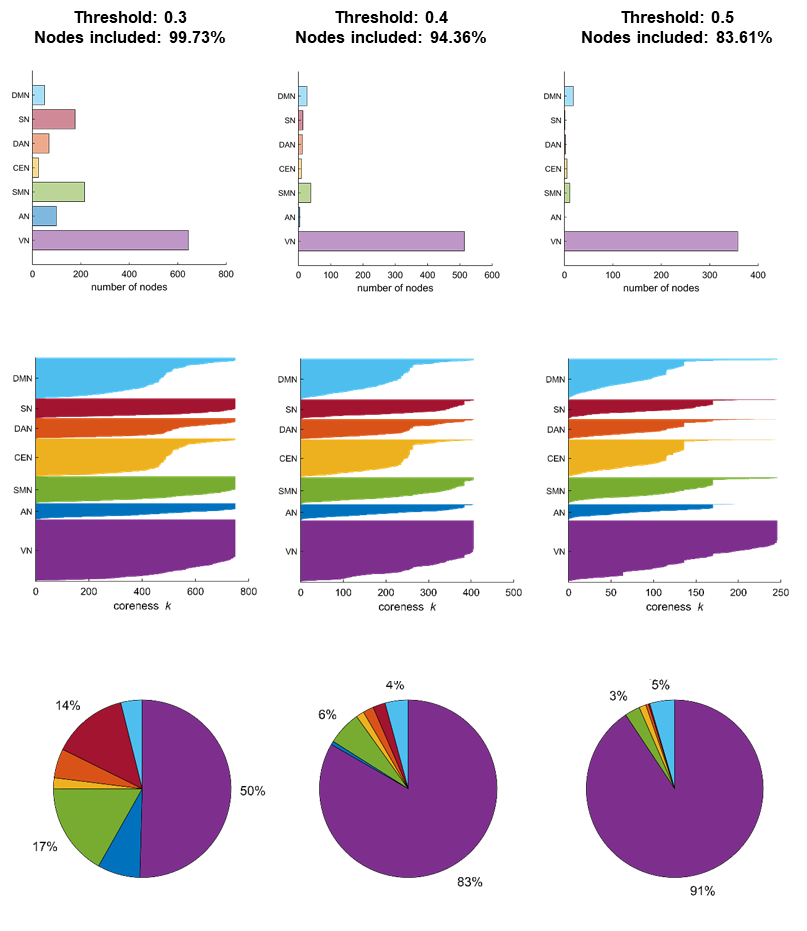


**Supplementary Figure 14.** The patterns of *k*_max_-core voxels when various thresholds were applied. We applied three thresholds: 0.3 (first column), 0.4 (second column), and 0.5 (third column). The percentages at the top indicate which nodes were included in the analysis at each threshold. The bar plots showing the number of *k*_max_-core voxels belonging to a specific independent component (IC) was displayed on the top. The flag plots show the change of the number of k-core voxels belonging to specific IC when k-core percolation is implemented (middle). The pie plots show the ratio of *k*_max_-core voxels belonging to each IC (bottom). This participant consistently shows the visual network (VN)-dominant pattern in all three thresholds.


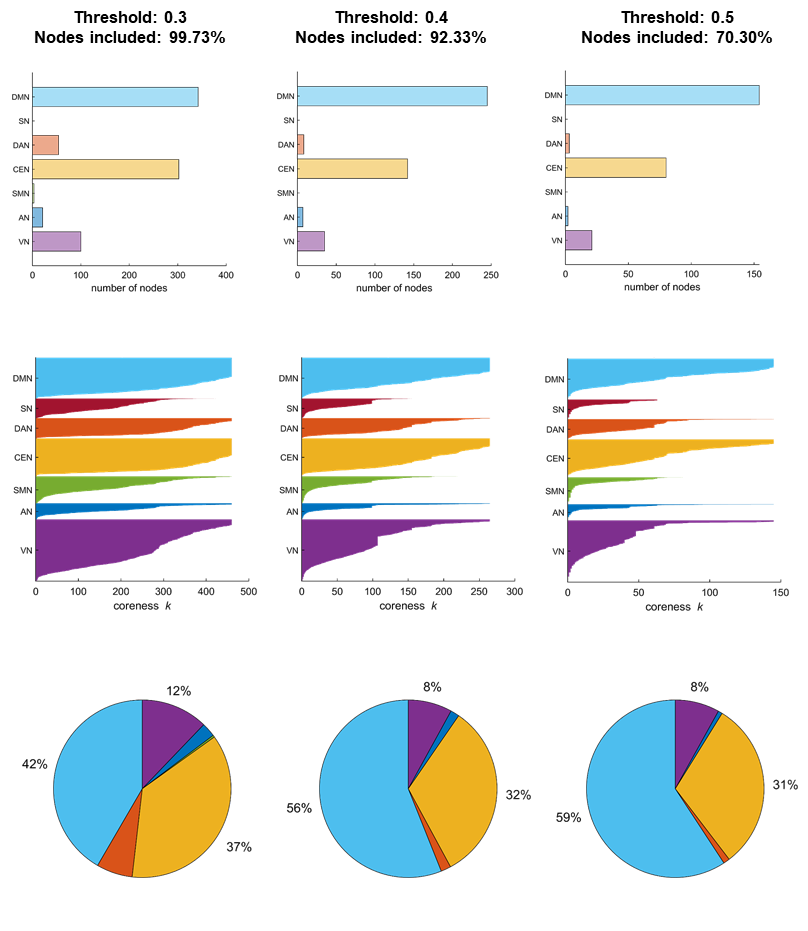


**Supplementary Figure 15.** The patterns of *k*_max_-core voxels when various thresholds were applied: 0.3 (first column), 0.4 (second column), and 0.5 (third column). The percentages at the top indicate which nodes were included in the analysis at each threshold. The bar plots (top), flag plots (middle), and pie plots (bottom) show that the participant consistently shows the DMN-dominant pattern in all three thresholds.


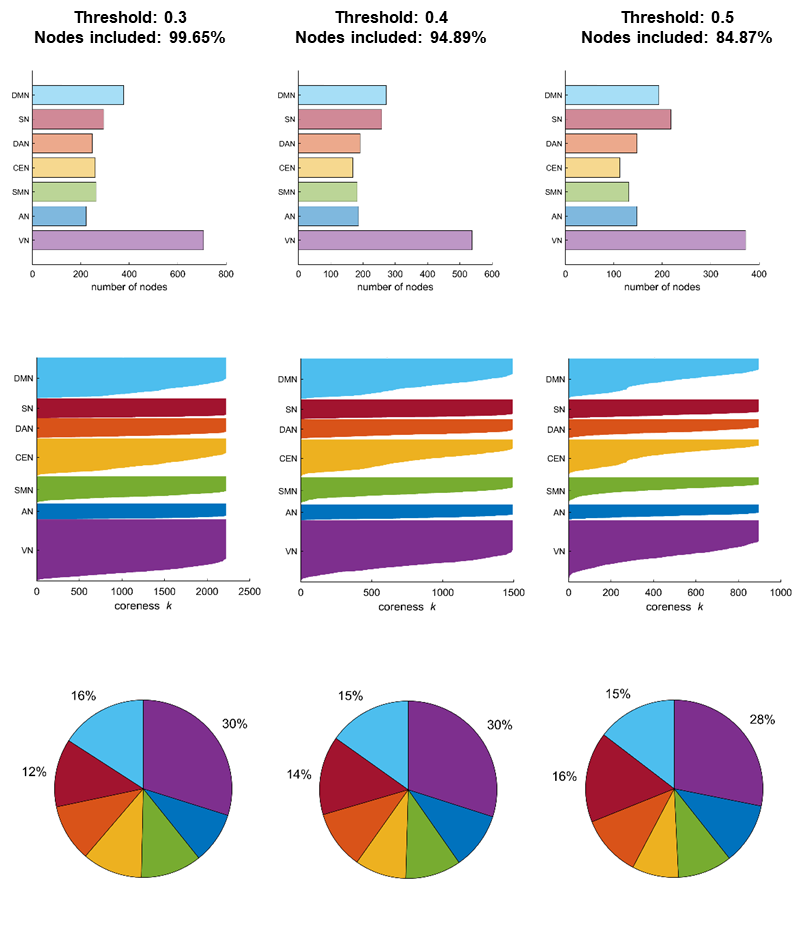


**Supplementary Figure 16.** The patterns of *k*_max_-core voxels were shown when various thresholds were applied: 0.3 (first column), 0.4 (second column), and 0.5 (third column). The percentages at the top indicate which nodes were included in the analysis at each threshold. The bar plots (top), flag plots (middle), and pie plots (bottom) show that the participant consistently shows the distributed pattern in all three thresholds.
